# Corona Immunitas: study protocol of a nationwide program of SARS-CoV-2 seroprevalence and seroepidemiologic studies in Switzerland

**DOI:** 10.1007/s00038-020-01494-0

**Published:** 2020-10-24

**Authors:** Erin A. West, Daniela Anker, Rebecca Amati, Aude Richard, Ania Wisniak, Audrey Butty, Emiliano Albanese, Murielle Bochud, Arnaud Chiolero, Luca Crivelli, Stéphane Cullati, Valérie d’Acremont, Adina Mihaela Epure, Jan Fehr, Antoine Flahault, Luc Fornerod, Irène Frank, Anja Frei, Gisela Michel, Semira Gonseth, Idris Guessous, Medea Imboden, Christian R. Kahlert, Laurent Kaufmann, Philipp Kohler, Nicolai Mösli, Daniel Paris, Nicole Probst-Hensch, Nicolas Rodondi, Silvia Stringhini, Thomas Vermes, Fabian Vollrath, Milo A. Puhan, the Corona Immunitas Research Group, Emiliano Albanese, Rebecca Amati, Antonio Amendola, Daniela Anker, Anna Maria Annoni, Andrew Azman, Frank Bally, Bettina Balmer, Hélène Baysson, Delphine Berthod, Jacob Blankenberger, Murielle Bochud, Patrick Bodenmann, Matthias Bopp, Audrey Butty, Anne Linda Camerini, Céline Cappeli, Cristian Carmelli, Arnaud Chiolero, Prune Collombet, Laurie Corna, Jenny Crawford, Luca Crivelli, Stéphane Cullati, Alexia Cusini, Valérie D’Acremont, Carlo De Pietro, Agathe Deschamps, Sophie Droz, Alexis Dumoulin, Olivier Duperrex, Julien Dupraz, Malik Egger, Nathalie Engler, Adina Mihaela Epure, Sandrine Estoppey, Marta Fadda, Vincent Faivre, Jan Fehr, Andrea Felappi, Maddalena Fiordelli, Antoine Flahault, Luc Fornerod, Cristina Fragoso Corti, Marion Frangville, Irène Frank, Giovanni Franscella, Anja Frei, Doreen Gille, Gisela Michel, Semira Gonseth Nusslé, Auriane Gouzowski, Idris Guessous, Julien Guggisberg, Huldrych Günthard, Felix Gutzwiller, Loussine Incici, Emilie Jendly, Ruedi Jung, Christian Kahlert, Laurent Kaiser, Laurent Kaufmann, Marco Kaufmann, Simone Kessler, Philipp Kohler, Susi Kriemler, Lauranne Lenoir, Sara Levati, Bettina Maeschli, Jean-Luc Magnin, Eric Masserey, Rosalba Morese, Nicolai Mösli, Natacha Noël, Maëlle Orhant, Jérôme Pasquier, Francesco Pennacchio, Dusan Petrovic, Stefan Pfister, Attilio Picazio, Cesarina Prandi, Giovanni Piumatti, Jane Portier, Nicole Probst-Hensch, Caroline Pugin, Milo Puhan, Thomas Radtke, Aude Richard, Claude-François Robert, Pierre-Yves Rodondi, Nicholas Rodondi, Eric Salberg, Javier Sanchis Zozaya, Virginie Schlüter, Valentine Schneider, Amélie Steiner-Dubuis, Silvia Stringhini, Johannes Sumer, Ismaël Tall, Julien Thabard, Mauro Tonolla, Nicolas Troillet, Agne Ulyte, Sophie Vassaux, Thomas Vermes, Fabian Vollrath, Viktor von Wyl, Erin West, Ania Wisniak, Maria-Eugenia Zaballa, Claire Zuppinger

**Affiliations:** 1 Epidemiology, Biostatistics and Prevention Institute, University of Zurich, Hirschengraben 84, 8001 Zurich, Switzerland; 2 Population Health Laboratory (#PopHealthLab), University of Fribourg, Fribourg, Switzerland; 3 Institute of Public Health, Università Della Svizzera Italiana, Lugano, Switzerland; 4 Unit of Population Epidemiology, Division of Primary Care Medicine, Geneva University Hospitals, Geneva, Switzerland; 5 Institute of Global Health, Faculty of Medicine, University of Geneva, Geneva, Switzerland; 6 Center for Primary Care and Public Health (Unisanté), University of Lausanne, Lausanne, Switzerland; 7 Institute of Primary Health Care (BIHAM), University of Bern, Bern, Switzerland; 8 Department of Epidemiology, Biostatistics, and Occupational Health, McGill University, Montreal, Canada; 9 Department of Business Economics, Health and Social Care, University of Applied Sciences and Arts of Southern Switzerland, Manno, Switzerland; 10 Department of Readaptation and Geriatrics, Faculty of Medicine, University of Geneva, Geneva, Switzerland; 11 Swiss Tropical and Public Health Institute, Basel, Switzerland; 12 University of Basel, Basel, Switzerland; 13 Health Observatory of Valais, Sion, Switzerland; 14 Clinical Trial Unit, Cantonal Hospital Luzern, Luzern, Switzerland; 15 Department of Health Sciences and Medicine, University of Luzern, Luzern, Switzerland; 16 Department of Infectious Diseases and Hospital Epidemiology, Cantonal Hospital St. Gallen, St. Gallen, Switzerland; 17 Infectious Diseases and Hospital Epidemiology, Children’s Hospital of Eastern Switzerland, St. Gallen, Switzerland; 18 Service de La Santé Publique, Canton de Neuchâtel, Neuchâtel, Switzerland; 19 Department of General Internal Medicine, Inselspital, Bern University Hospital, University of Bern, Bern, Switzerland; 20 Corona Immunitas Program Management Group, Swiss School of Public Health, Zurich, Switzerland

**Keywords:** Prevalence, Serosurvey, Longitudinal, SARS-CoV-2, Socioeconomic differences, Hygiene practices

## Abstract

**Objectives:**

Seroprevalence studies to assess the spread of SARS-CoV-2 infection in the general population and subgroups are key for evaluating mitigation and vaccination policies and for understanding the spread of the disease both on the national level and for comparison with the international community.

**Methods:**

Corona Immunitas is a research program of coordinated, population-based, seroprevalence studies implemented by Swiss School of Public Health (SSPH+). Over 28,340 participants, randomly selected and age-stratified, with some regional specificities will be included. Additional studies in vulnerable and highly exposed subpopulations are also planned. The studies will assess population immunological status during the pandemic.

**Results:**

Phase one (first wave of pandemic) estimates from Geneva showed a steady increase in seroprevalence up to 10.8% (95% CI 8.2–13.9, *n* = 775) by May 9, 2020. Since June, Zurich, Lausanne, Basel City/Land, Ticino, and Fribourg recruited a total of 5973 participants for phase two thus far.

**Conclusions:**

Corona Immunitas will generate reliable, comparable, and high-quality serological and epidemiological data with extensive coverage of Switzerland and of several subpopulations, informing health policies and decision making in both economic and societal sectors.

ISRCTN Registry: https://www.isrctn.com/ISRCTN18181860.

**Electronic supplementary material:**

The online version of this article (10.1007/s00038-020-01494-0) contains supplementary material, which is available to authorized users.

## Introduction

By August 21, 2020, over 22,000,000 persons were diagnosed with a severe acute respiratory syndrome coronavirus-2 (SARS-CoV-2) (Gorbalenya et al. 2020) infection globally and the number of deaths exceeded 790,000 (Worldometer 2020). Switzerland alone had over 39,000 confirmed cases with over 1700 deaths (Federal Office of Public Health (FOPH) 2020a). The 2019 novel coronavirus (COVID-19) pandemic has caused worldwide lockdowns and restrictions on individuals’ freedom of movement to limit the spread of this virus. The measures taken by the Swiss government aimed to balance the protection of the population against SARS-CoV-2 and the functioning of the economy and society at large. As the number of cases rose in March 2020, a containment strategy of contact tracing and isolation was not enough (Hellewell et al. 2020) and the country was quickly put into a semi-lockdown. Non-essential businesses were closed, citizens were asked to stay at home, and distance learning for all schools was implemented (FOPH 2020b). Later, with the flattening of the pandemic curve and increasing political pressure, the semi-lockdown was progressively lifted. These decisions had, and still have, to be taken, while many epidemiologic features of the SARS-CoV-2 pandemic are unknown, such as transmission characteristics of the virus, prevalence of infection, extent of immunity after infection and its relationship to disease symptoms and severity, and how different groups of the population are affected. To overcome this, it is crucial to monitor the pandemic and increase our knowledge about the virus with solid data to inform future policy decisions and help prepare potential future outbreak responses in a balanced manner (Arora et al. 2020).

Seroprevalence surveys are designed to assess the proportion of populations infected by the virus, where diagnostic testing fails to ascertain the full scale of the spread due to unreported and asymptomatic cases. However, weaknesses and between-study differences in methodology limit their reliability and comparability. A rapid, systematic review of early seroprevalence surveys by Bobrovitz et al. (preprint) on SARS-CoV-2 came out identifying 73 completed and ongoing seroprevalence studies across the world. These studies reported seroprevalence estimates ranging between 0.4 and 59.3%. The review found that, of the 23 studies reporting prevalence estimates, many had a moderate or high risk of bias (Bobrovitz et al. 2020; Joanna Briggs Institute 2016). Reportedly, the largest issues with these early studies included inadequate sampling methods and antibody test performance, lack of or inconsistent use of questionnaire assessments, and varying designs and analyses, hindering comparison of results.

To overcome these limitations and generate information about the SARS-CoV-2 pandemic that is accurate and generalizable to the whole population, population-based seroprevalence studies with reliable methodologies are needed (Pollán et al. 2020). It is essential to include strong coordination across study sites and to ensure cross-regional comparability. Standardized methodologies, including the same antibody tests, questionnaires, and study procedures, are vital to obtain reliable estimates within a country (World Health Organization (WHO) 2020). Further, an additional understanding of the duration of immunity, demographic differences, and the economic and psychological impact of the pandemic and subsequent control measures adds more depth and understanding of the overall situation. This further helps inform policy-making and provides better preparedness, both logistically and with respect to the economic consequences, for any future outbreaks.

We describe here the protocol of Corona Immunitas, a centrally coordinated research program consisting of repeated cross-sectional and longitudinal seroprevalence and seroepidemiological studies conducted across several regions and populations in Switzerland, whose aim is to generate reliable data to inform policy-making.

### Study objectives

The main goal of the Corona Immunitas research program is to determine the extent and nature of infection with SARS-CoV-2 in Switzerland in a highly consistent and comprehensive way, first, in the general population and, second, in vulnerable and highly exposed subpopulations. Specific aims are to: (1) estimate the number of individuals infected with SARS-CoV-2 in the population with or without symptoms at several points in time; (2) compare the seroprevalence between the general population and specific subpopulations; (3) investigate the characteristics, duration, and extent of immunity after infection; (4) assess the association between participant characteristics and behaviors with their risk of infection; and (5) quantify the association between the pandemic and participants’ mental and physical health. Most importantly, this evidence-based program aims to provide policy-makers and other decision makers with important evidence for deciding which public health and setting-specific measures to implement or lift in the general population and specific subpopulations at different points in time.

## Methods

The protocol has been developed according to the Consortium for the Standardization of Influenza Seroepidemiology (CONSISE) Statement on the Reporting of Seroepidemiologic Studies for Influenza (ROSES-I) (Joanna Briggs Institute 2016).

### Study design and phases of the Corona Immunitas program

The Corona Immunitas program includes more than 20 cross-sectional and longitudinal studies in the general population and in specific subpopulations (Tables 1 and 2) with serological testing at baseline and a digital only or combined digital and serological follow-up. The questionnaires and the antibody test are standardized across the sites to guarantee comparability across the country. The Corona Immunitas research group shares protocols for all the studies in an open science way in order to exchange knowledge and expertise, to create synergisms but also to reduce redundancies across cantons and regions. We define our general, population-based studies as seroprevalence studies and our subpopulation studies as seroepidemiological studies per the lexicon defined by Horby et al. (2017).

**Table 1:** Characteristics of the population-based seroprevalence studies by study center (Corona Immunitas, Switzerland, 2020–2021)

Region	Setting				Aimed sample size			Laboratory methods		Biobank for long-term storage	
Study centers, city, cantons covered by the study (name of the study)	Sampling method*	*n* cross-sectional samples (recruitment period)	Age groups (yrs)	Longitudinal component**	Per age group	Per sample	Total	Quantity of blood collected (ml)	Sample type	Storage temperature and duration	Consent for genetic analyses
Geneva University Hospital, Geneva, GE, Switzerland, 2020 (SEROCoV-POP)	Random sampling from participants in former study (de Mestral et al. 2019) +household members	12 (Apr to Jun)	5+	Yes	N/A	N/A	8640	1.5–3 (age dependent)	Serum, no preparation	− 80 °C for max 30 yrs	No
Unisanté, Lausanne, VD, Switzerland, 2020 (SérocoViD)	Standard*	6 (s1: May; s2: Nov)	0.5–4 5–9 10–14 15–19 20–39 40–64 65–74 75+	Yes	100	800	4800	2.5–25 (age dependent)	Serum and plasma	− 80 °C for 20 years or unlimited time (depending on consent)	Yes for ≥ 14-year-olds
Population Health Laboratory, Fribourg, FR, Switzerland, 2020 (Corona Immunitas Fribourg)	Standard*	2 (s1: Jul, Aug s2: Nov)	20–64 65+	No	300	600	1200	7.5	Serum	N/A	N/A
Swiss TPH, Basel, BS and BL, Switzerland, 2020 (COVCO-Basel)	Age-stratified and half-canton-stratified random subsamples of digital cohort; digital cohort was sampled through standard* procedure	2 (Jun and Sep)	18–49 50–64 65+ + family members	Yes	200	600	1200 (+ 800 family members)	10–20 (age dependent)	Serum, EDTA	− 80 °C for 10 yrs or unlimited (depending on consent)	Yes
Cantonal Medical Service, Neuchâtel, NE, Switzerland, 2020 (Corona Immunitas Neuchâtel)	Standard*	2 (s1: Jul; s2: Nov)	20–64 65+	No	300	600	1200	12	Serum	− 80 °C for 5 yrs	No
Cantonal hospital and Children’s Hospital of Eastern Switzerland, St. Gallen, SG and GR (Corona Immunitas Eastern Switzerland)	Standard*	1 (Sep)	5–19 20–64	Yes	300	750	1500	10	Serum	N/A	N/A
Institute of Public Health and Department of Business Economics, Health & Social Care, Lugano, TI, Switzerland, 2020 (Corona Immunitas Ticino)	Standard* + family members aged 5–20 and 65+ yrs in s2	2 (s1: Jul and s2: Nov)	s1: 20–44 45–64 s2: 5–9 10–13 14–19 65–74 75+	Yes	s1: 200–500 s2: no target *n* per age subgroup (*n* = 400–2000 For family members aged < 20 and 65+ yrs)	s1: 400–1000 s2: 800–2000 + 400–2000 family members	1600–5000	8.5	Serum	− 20 °C and − 80 °C for 1–2 yrs	No
Epidemiology, Biostatistics and Prevention Institute, Zurich, ZH, Switzerland, 2020 (Corona Immunitas Zurich)	Standard*	2 (s1: Jun, Aug; s2: Nov)	s1: 20–44 45–64 65+ s2: 20–64 65+	Yes	s1: 20–44 yrs, *n* = 200 45–64 years, *n* = 200 65+ yrs, *n* = 400 s2: 20–64 yrs, *n* = 200 65+ yrs, *n* = 200	s1: 800 s2: 400	1200	20	Plasma	− 80 °C for 5 yrs	Unclear
Valais Health Observatory & Central Hospital Institute, Valais Hospital, Sion, VS (Corona Immunitas Valais/Wallis)	Standard*	1 (Oct)	20–64 65+	No	600 (3 regions; 200/region)	1200 (3 regions; 400/region)	1200	CND	CND	CND	No
Institute of Primary Health Care (BIHAM), Bern, BE, Switzerland, 2020 (Corona Immunitas Bern)	Standard*	CND	20–64 65+	No	300	600	1200	7.5	Serum	N/A	N/A
Clinical Trial Unit (CTU), Luzern Cantonal Hospital, LU, Switzerland, 2020 (Corona Immunitas Luzern)	Standard*	CND	20–64 65 +	No	300	600	1200	7.5	Serum	N/A	N/A

All months are in 2020. Items in this table are based on CONSISE statement on the reporting of Seroepidemiologic Studies for influenza (10). N/A, not applicable; yrs, years; s1-12, sample 1–12; *n*, number; GE, Canton Geneva; VD, Canton Vaud; FR, Canton Fribourg; BS, Canton Basel-City; BL, Canton Basel-Land; NE, Canton Neuchâtel; SG, Canton St. Gallen; TI, Canton Ticino; ZH, Canton Zurich; VS, Canton Valais; BE, Canton Bern; LU, Canton Lucerne; CND, currently not defined; SérocoViD, Understanding community transmission and herd immunity related to SARS-CoV-2 in the Canton of Vaud to inform public health decisions, Switzerland, 2020; CoV-Co-Basel, Population-based SARS-CoV-2 Cohort Basel-Land and Basel-City, Switzerland, 2020; CTU, Clinical Trial Unit of Luzern Cantonal Hospital; BIHAM, Bern Institute for Family Medicine. (Corona Immunitas, Switzerland, 2020–2021)

*Standard sampling method: random sampling from residential registry of each Canton stratified by pre-defined age groups

**Longitudinal component: refers to whether there are several blood collections for serology in the same individuals

**Table 2:** Characteristics of specific subpopulations investigated by study center (Corona Immunitas, Switzerland, 2020–2021)

Location	Subpopulation and aim		Setting		
Study center, city, canton covered by the study	Subpopulation investigated (name study)	Specific aim	Study design (time points and setting of data collection)*	Sampling and aimed sample size	Recruitment period
Geneva University Hospital, Geneva	Several studies, independently funded, designed by different investigators	N/A	N/A	N/A	N/A
Unisanté, Lausanne, VD	Confirmed COVID-19 cases and their household or otherwise close contacts (SérocoViD)	Investigate SARS-CoV-2 transmission in the community, including: Radius and influencing factors of transmission, Proportion of asymptomatic and pauci-symptomatic individuals, Characteristics of confirmed COVID-19 cases and seropositive close contacts	Cross-sectional seroprevalence study	200 symptomatic COVID-19 patients with positive RT-PCR registered during first 5 weeks of the pandemic in the cantonal registry, including: Index cases of the first 10 days (*n* = 20) Randomly selected cases in weeks 2, 3, 4, and 5 (*n* = 120) All cases of children (*n* = 60) + all close contacts identified through the active tracing	Apr
	Employees in following sectors: food retailer public transportation post office laundry services (SérocoViD)	Seroprevalence in employees working in highly exposed sectors due to proximity to customers or other employees	Cross-sectional seroprevalence study	Random sampling from all employees in predefined sectors. Number invited: Food retailer, *n* = 240 Public transportation, *n* = 209 Post office, *n* = 310 Laundry services, *n* = 245	May
	Asylum seekers (SérocoViD)	Seroprevalence in asylum seekers constrained to live in the same home in high numbers	Cross-sectional seroprevalence study	Two centers of similar size and location: one with many reported COVID-19 cases and one with few	May
Population Health Laboratory, Fribourg	None	N/A	N/A	N/A	N/A
Swiss TPH, Basel, BS and BL	Family members of participants in the population-based seroprevalence study (Table 1)	Compare seroprevalence and mental health, well-being, behavior within families	Longitudinal seroprevalence study	2 samples of 400 family members of participants in the population-based study (total *n* = 800)	Jul–Sep, Oct–Dec
Cantonal hospital and Children’s Hospital of Eastern Switzerland, St. Gallen, SG and GR	Hospital employees from healthcare institutions in Eastern Switzerland (SURPRISE)	Seroprevalence, symptoms and risk factors for COVID-19 among healthcare workers	Longitudinal seroprevalence study (BL + 1 FU)	All employees of the Cantonal hospital St. Gallen aged 16 yrs and more are invited, target *n* = 5000–15,000	Jul and Nov
	Workers at the children’s hospital “Ostschweizer Kinderspital” (CIMOKS)	Investigate seroconversion for SARS-CoV-2 among workers of a children’s hospital	Cross-sectional seroprevalence study (capillary blood collection)	Weekly random samples of 50 German-speaking workers of the children’s hospital	Jun and Sep
Institute of Public Health and Department of Business Economics, Health & Social Care, Lugano, TI	Healthcare workers (SARS-CoV-2)	Seroprevalence by level of risk of contagion across health services in healthcare workers. Level of risk is based on site (COVID-19 or non-COVID-19 dedicated clinic), ward, and profession, e.g., medical vs administrative staff	Longitudinal seroprevalence study (BL + 2 FU or more)	All healthcare workers of the cantonal hospitals (*n* = 4334) and two clinics (*n* = 394)	May and July
	Nursing home healthcare workers (COV-RISK)	Seroprevalence	Longitudinal seroprevalence study (serology: BL + 3 FU; questionnaires: monthly)	All healthcare workers in selected nursing home (convenience selection; *n* = 900)	Jul
	Nursing home residents (COV-RISK)	Seroprevalence and psychological impact in nursing home residents	Longitudinal seroprevalence study (BL + 3 FU; capillary blood is optional)	All residents in selected nursing home (convenience selection; *n* = 900)	Jul
	Inter-generational household contacts/family	Secondary infection rate, secondary attack rate	Cross-sectional seroprevalence study	*n* = 400–1000 participants < 20 yrs of sample 2 of the population-based study + up to 1000 family members (65+ yrs) *n* = 400–1000 participants 65+ yrs of sample 2 of the population-based study + up to 1000 family members (< 20 yrs)	Sep
	Inter-generational household contacts/family	Rate of re-infection and duration of acquired immunity	Longitudinal nested case–control study (BL + 3 FU; BL is part of the population-based study in Table 1)	N total = 200 case–controls from sample 2 of the population-based study (Table 1): Positive cases defined based on seropositivity: < 20 yrs, *n* = 50; 65+ yrs, *n* = 50 Matched negative controls: < 20 yrs, *n* = 50; 65+ yrs, *n* = 50	Sep
Epidemiology, Biostatistics and Prevention Institute, Zurich, ZH	Spitex employees	Seroprevalence in particularly exposed population	Cross-sectional seroprevalence study	All Spitex employees (convenience selection)	Jul
	Employees of nursing homes	Seroprevalence in particularly exposed population	Cross-sectional seroprevalence study	All employees of nursing homes (convenience selection)	Jul
	Persons who receive opioid agonist therapy (substitutions)	Seroprevalence in persons who receive opioid agonist therapy (substitutions)	Cross-sectional seroprevalence study	All persons receiving therapy at Arud Center for Addiction Medicine (convenience selection)	Jul
	Individuals participating in the SwissPrEPared study	Seroprevalence in persons who take pre-exposure HIV prophylaxis and participate in the SwissPrEPared study	Cross-sectional seroprevalence study	All participants of SwissPrEPared (convenience selection; *n* = 2067)	Jul
	Persons who contacted the COVID-19-Test Center of the UZH for a SARS-CoV-2 antibody test because of symptoms suggestive of COVID-19	Seroprevalence persons with proactive request for SARS-CoV-2 antibody test	Cross-sectional seroprevalence study	All who request a test at the COVID-19 Test Center (convenience selection)	Jul
	School children, attending grades 1–8, i.e., approximate age 5–16 years old, in a public or private school + parents and school employees of the selected schools (Ciao Corona)	Seroprevalance and its temporal changes, clustering of cases within classes, schools and districts, symptoms, and risk factors in a representative cohort of children and adolescents shortly after reopening of the school system and thereafter	Longitudinal seroprevalence study (serology: BL + 2 FU, questionnaire: monthly; school principals followed-up with monthly questionnaires only)	Random sampling on school level, grade level, and class level. Target sample size: Primary school children, *n* = 1700 Secondary school children, *n* = 850 Parents, *n* = 2500–5000 School employees, *n* = 1500–3000	Jun (children) and Aug (adults)
Valais Health Observatory & Central Hospital Institute, Valais Hospital, Sion, VS	None	N/A	N/A	N/A	N/A
Institute of Primary Health Care (BIHAM), Bern, BE	None	N/A	N/A	N/A	N/A
Clinical Trial Unit (CTU), Luzern Cantonal Hospital, LU	None	N/A	N/A	N/A	N/A

All months are in 2020. Items in this table are based on CONSISE statement on the reporting of Seroepidemiologic Studies for influenza (ROSES-I statement) (10). N/A, not applicable; yrs, years; s1-12, sample 1–12; *n*, number; BL, baseline; FU, follow-up; GE, Canton Geneva; VD, Canton Vaud; FR, Canton Fribourg; BS, Canton Basel-City; BL, Canton Basel-Land; NE, Canton Neuchâtel; SG, Canton St. Gallen; TI, Canton Ticino; ZH, Canton Zurich; UZH, University of Zurich; VS, Canton Valais; BE, Canton Bern; LU, Canton Lucerne; SérocoViD, Understanding community transmission and herd immunity related to SARS-CoV-2 in the Canton of Vaud to inform public health decisions, Switzerland, 2020; CoV-Co-Basel, Population-based SARS-CoV-2 Cohort Basel-Land and Basel-City, Switzerland 2020; SURPRISE, Severe AcUte Respiratory Syndrome Coronavirus-2 among Healthcare Professionals In Switzerland, Switzerland, 2020; CIMOKS, Corona immunity for employees of the Children's Hospital of Eastern Switzerland, Switzerland, 2020; CTU, Clinical Trial Unit of Luzern Cantonal Hospital; BIHAM, Bern Institute for Family Medicine. (Corona Immunitas, Switzerland, 2020–2021)

*Data collection involves both blood sample collection for serology and questionnaire unless stated otherwise

**Fig. 1:**
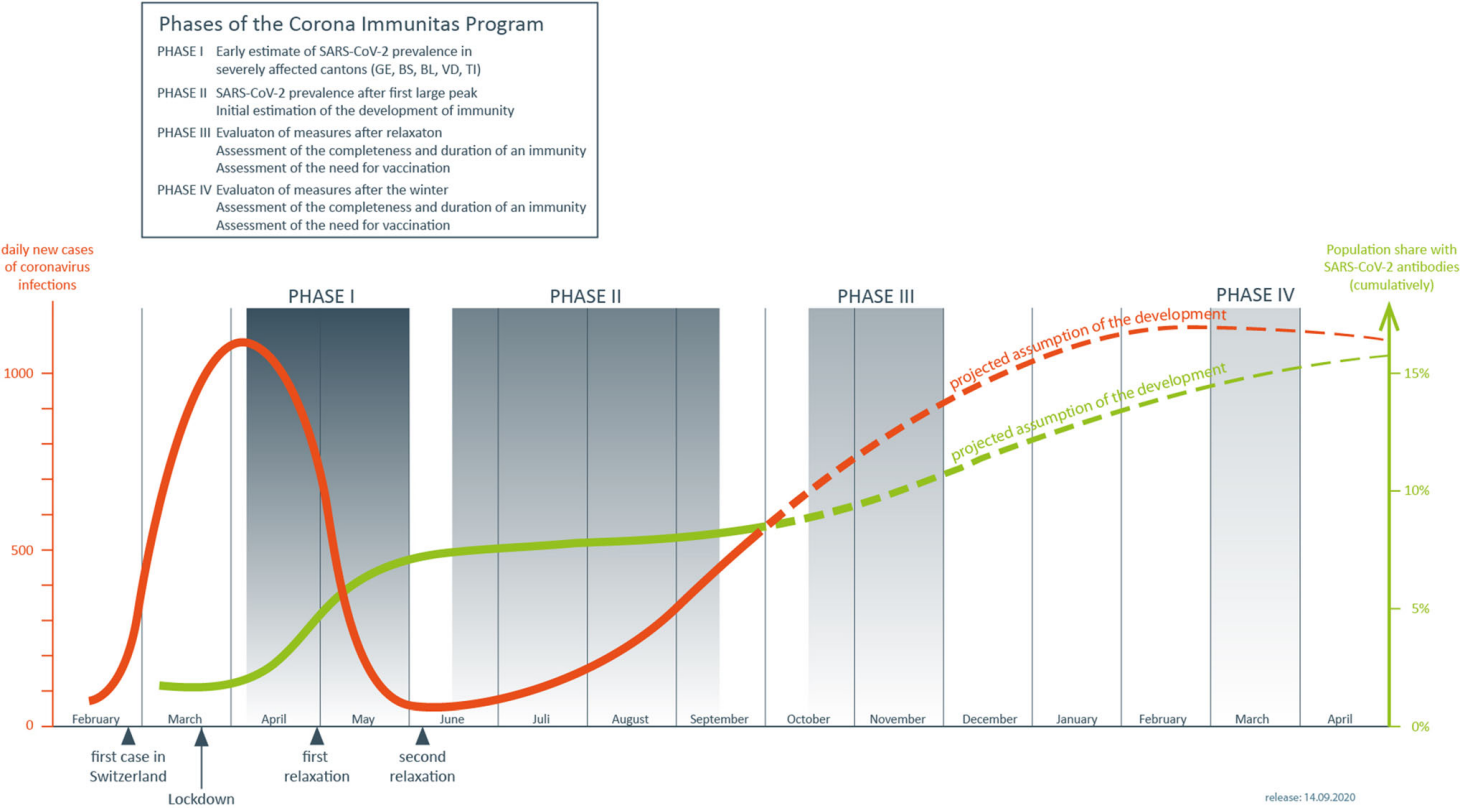
Phases of the pandemic as defined in the Corona Immunitas program (Switzerland 2020–2021)

The studies are conducted in a series of repeated phases, the timing of which is subject to change according to the highly dynamic pandemic and additional needs that may emerge (Fig. 1). The first phase began in April 2020 and included early estimates of seroprevalence during the first wave from Geneva (Stringhini et al. 2020). The second phase includes estimates of seroprevalence across Switzerland during summer, after the first peak of the pandemic. This phase, conducted as recommended by the WHO protocol (WHO 2013) after the (first) pandemic wave, includes highly (Cantons of Geneva, Vaud and Ticino), moderately (Cantons of Fribourg, Neuchatel and Basel City/Land) and little affected regions of Switzerland (Cantons of Zurich and St. Gallen) (FOPH 2020a). The third phase will be in the fall and includes estimates of seroprevalence across Switzerland about 4–5 months after the lifting of lockdown measures. This phase will help evaluate the quality of the monitoring systems in place and determine the needs for a vaccination program. A fourth phase will cover March 2021 and will evaluate the measures after the winter, as well as the completeness and duration of immunity.

### General population seroprevalence studies

Geneva was the first canton to enroll randomly selected participants of a population-based study (Bus Santé) ongoing since 1993 (Stringhini 2020). For the other Cantons, the Federal Statistical Office (FSO) randomly selected, per region, potential participants from the residential registry. The number of randomly chosen participants takes into consideration the net sample size (i.e., target sample size) as well as the expected participation rate and reserve samples (residents selected in the case that more participants are needed to fulfill the sample size requirements). The FSO provides all participating Cantons with a list of participants, including their full name, postal address and preferred language. The sampling is age-stratified into three age groups: 5–19 years, 20–64 years, and 65 years and older, though some Cantons have also chosen to recruit younger participants. Cantons can choose slightly modified sampling procedures, such as recruiting participants’ family or household members (Table 1).

The inclusion criteria for the random sampling are residency in one of the participating Cantons. “Vulnerable to COVID-19 persons” (detailed below) are included. For inclusion in the data collection, individuals with a suspected or confirmed acute SARS-CoV-2 infection are postponed for a visit to 48 h after disappearance of symptoms or 21 days after a positive SARS-CoV-2 reverse transcription polymerase chain reaction test (RT-PCR) result. Excluded from the residential registry of the FSO are diplomats, persons with a foreign address in the registry, persons in asylum procedure, persons with a short-term residence permit, and elderly people in nursing homes.

If selected, “vulnerable to COVID-19 persons,” i.e., persons at risk of a severe course of SARS-CoV-2 infection, can participate. They are defined in accordance with the recommendations by the Swiss Federal Office of Public Health (FOPH) (FOPH 2020c). The handling of those participants vulnerable to COVID-19 varies depending on the site. Persons are considered at risk of a severe course of SARS-CoV-2 infection if they are 65 years of age or older, pregnant or if they have the following conditions: diabetes; cardiovascular disease; chronic diseases of the respiratory tract; immune weaknesses due to disease or therapy; cancer(s); and obesity defined by a body mass index (BMI) > 30 kg/m^2^ for adults and above the 97th percentile of Swiss BMI growth curves for children and adolescents (https://www.paediatrieschweiz.ch). While the FOPH considers persons with high blood pressure to be at risk, the Swiss Hypertension Society recommends applying the same precautionary measures for hypertensive patients as for the rest of the population (Swiss Society of Hypertension 2020).

### Subpopulation seroepidemiological studies

**Fig. 2:**
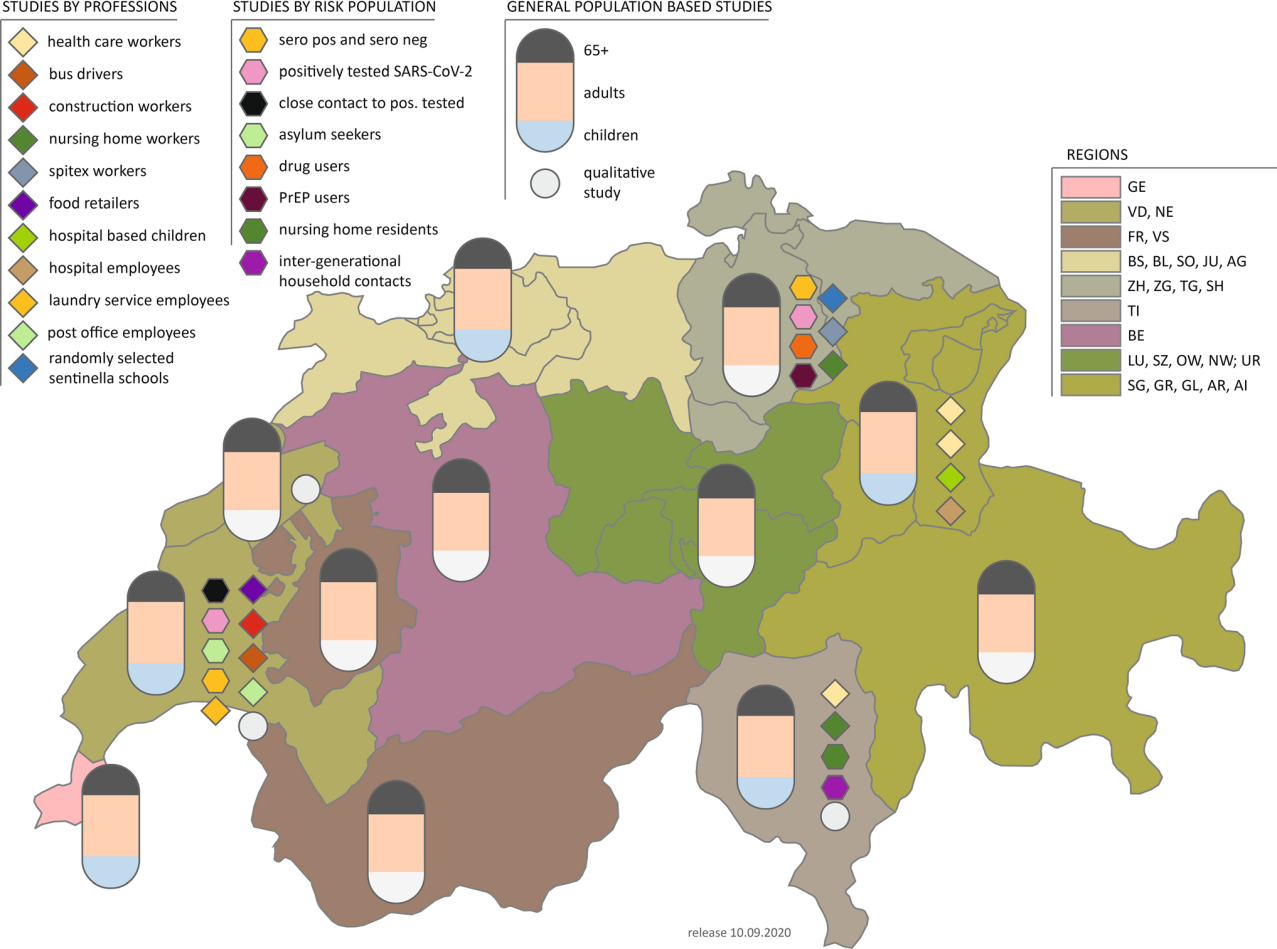
Overview of seroprevalence studies in Switzerland (Corona Immunitas 2020–2021). *ZH* Zurich, *BE* Bern, *LU* Luzern, *UR* Uri, *SZ* Schwytz, *OW* Obwald, *NW* Nidwald, *GL* Glarus, *ZG* Zug, *FR* Fribourg, *SO* Solothurn, *BS* Basel-City, *BL* Basel-Land, *SH* Schaffhausen, *AR* Appenzell Ausserrhoden, *AI* Appenzell Innerrhoden, *SG* St. Gallen, *GR* Graubünden, *AG* Aargau, *TG* Thurgau, *TI* Ticino, *VD* Vaud, *VS* Valais, *NE* Neuchâtel, *GE* Geneva, *JU* Jura, *PrEP* Pre-exposure prophylaxis for HIV prevention. See Tables 1 and 2 for a detailed description of the studies. (Corona Immunitas, Switzerland, 2020–2021). *Children study with white bottoms instead of blue bottoms means there is no population-based children’s study

Corona Immunitas also includes specific subpopulations deemed especially exposed or vulnerable and about which more knowledge is necessary to inform policy- and decision making. Each center decided which subpopulation should be targeted based on the needs of stakeholders (e.g., Cantonal health authorities) or scientific interest. These studies follow the same study protocol as the general population but may include additional questions (e.g., characteristics or specific participant-reported outcomes). Figure 2 and Table 2 show a comprehensive overview of these subpopulation studies, notably nursing home residents and staff, healthcare workers, school students, teachers, and parents.

### Study recruitment and informed consent

Participants of the general and subpopulation studies are invited to participate by postal mail or email. The invitation requests that interested participants make an appointment for a baseline assessment and contains information about the study, a declaration of consent, and an electronic link that will allow them to complete a baseline questionnaire online. Feedback of the serology test to participants is handled individually by the sites and subject to variations.

### Baseline assessment

**Fig. 3:**
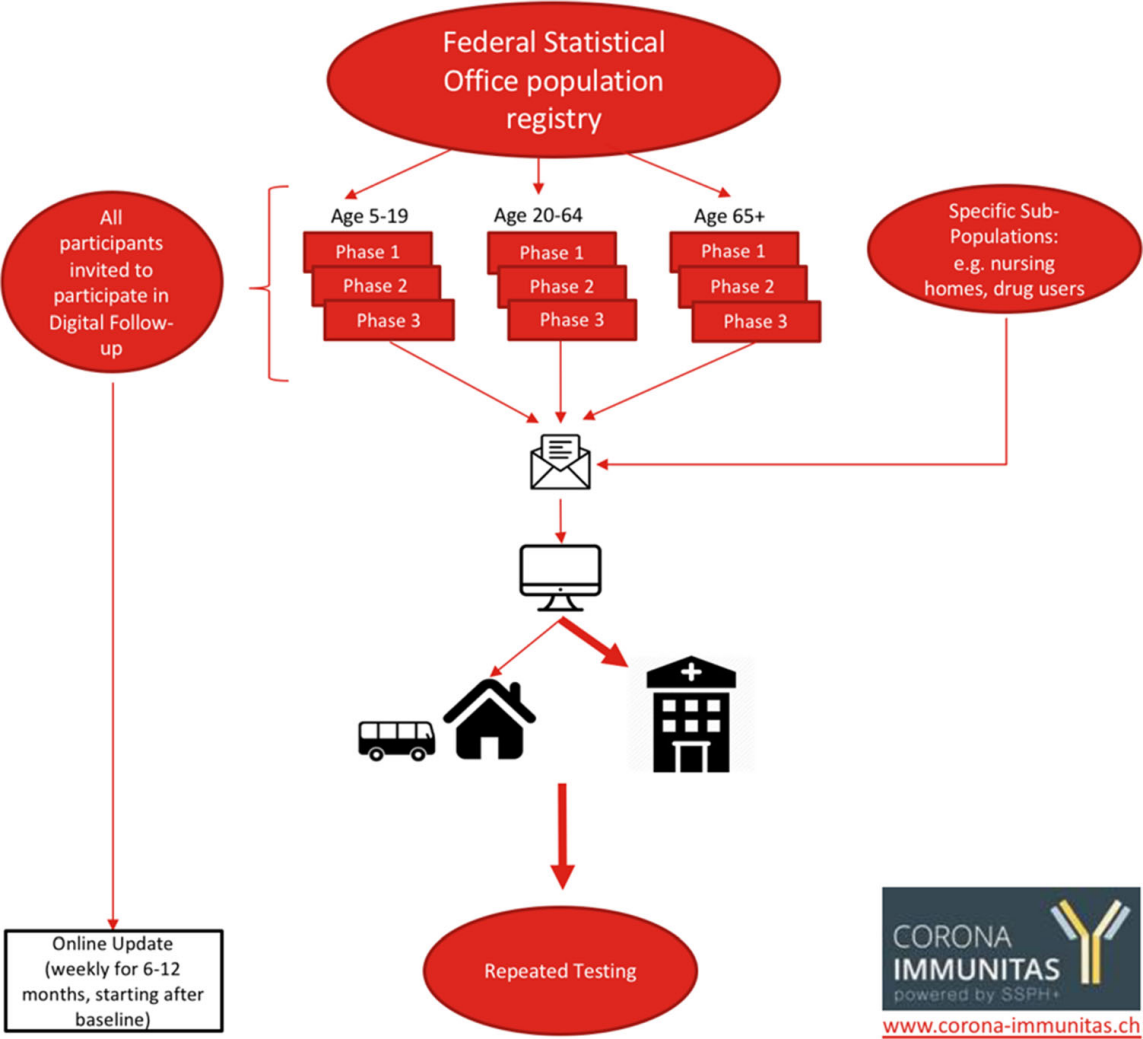
Example study flow of seroprevalence studies (Corona Immunitas, Switzerland, 2020–2021)

An example of the full study flow is outlined in Fig. 3. Informed written or electronic consent is obtained before any procedure of the study visit. Participants can fill the baseline questionnaire online or use a paper form. The questionnaire takes approximately 20 min to complete and includes demographic questions, symptoms, other tests taken for SARS-CoV-2, preventative measure behaviors, and quality of life measures. Details of the questions asked of the participants are given in Table 3. The full questionnaires used nationwide are published in the online supplementary material.

**Table 3:** Example of schedule of assessments (Corona Immunitas, Switzerland, 2020–2021)

Contacts	Invitation/recruitment	Study visit (BL)	Digital follow-up
Timing	Prior to Day 0	Day 0	Week 1 to week 52 (weekly)
Study invitation	X		
Invitation for Baseline data collection (and visit—depending on center)	X		
Confirmation letter or email, written information	X		
Baseline questionnaire (online): Personal demographics & health data COVID-19 specific data; including symptoms, hospitalization, relevant medications, other SARS-CoV-2 tests Socio-demographic data Economic impact due to lockdown measures Persons in immediate vicinity and their relevant symptoms Preventive measures, exposure and level of concern over pandemic	X	X	
Oral and/or written information		X	
Check inclusion-/ exclusion criteria		X	
Written consent		X	
Blood sampling		X	
Information on test interpretation		X	
Information on digital follow-up		X	
Information on further procedure according to subpopulation		X	
Weekly digital follow-up questionnaire (online): Symptoms, healthcare professional contacts, hospitalizations, test results, preventive measures			X
Monthly digital follow-up questionnaire (online): Access to health care, behavior, daily activities, mental health, well-being, usage of SwissCovid App (FOPH 2020d), motivation to participate in study			X

*BL* baseline

Venous blood sampling is performed at a blood collection site or at home. All staff have access to the necessary infrastructure for blood withdrawal and safe storage of biological samples. The entire process follows Standardized Operating Procedures (SOPs). Blood drawing is performed by trained healthcare staff (i.e., nurse, assistant nurse, or junior doctor). The quantity of blood varies according to the study site, and depending on whether additional analyses are performed. Standard hygiene rules are followed such as usual handwashing, disinfection procedures, and wearing of masks and gloves. All participants are required to wear a mask, provided by the study team, during all interactions.

Samples are transported to a local laboratory or the Vaud Central University Hospital (CHUV) directly or centrifuged before transport to the laboratory if possible, aliquoted and stored in a biobank on each site at − 20 °C or − 80 °C before the serological test. Samples are delivered within 16 h of being taken. Team members are trained in safe management practices and procedures for contamination accidents. Serum is prepared with serum gel and plasma with ethylenediaminetetraacetic acid (EDTA) before the serology test. Depending on the site, serum or plasma from the drawn venous blood is analyzed for the presence of SARS-CoV-2 IgG and IgA antibodies. Some study sites will store additional serum, saliva or plasma samples in a biobank for further use in this or in other studies; genetic and epigenetic analyses are planned in several centers. Participants are informed about the planned analyses and provide broad consent for future research use of biospecimens.

The study data are collected and managed using REDCap electronic data capturing tools (Harris et al. 2019) hosted at the responsible universities. Websites of study centers are fully detailed in the supplementary material Table S1.

### Selection of antibody tests

The selection of a common test followed a stepwise procedure. We developed a set of criteria (see supplementary material Table S2) that refer to the nature of the test, results from validation studies and the logistics and cost of the test. Members of the Corona Immunitas consortium independently rated the tests that were submitted for use in our program, and compiled a ranking (Corona Immunitas 2020). While specificity was high for most tests, which is crucial when seroprevalence is low, there was evidence of limited sensitivity. Most validation studies are likely to be substantially biased (e.g., spectrum bias and differential verification bias) because of their designs according to recent systematic reviews (Corona Immunitas 2020; Deeks et al. 2020). Biased estimates of accuracy make the adjustment of seroprevalence estimates for (imperfect) sensitivity and specificity uncertain. Therefore, the members decided to use the SenASTrIS (Sensitive Anti-SARS-CoV-2 Spike Trimer Immunoglobulin Serological) assay developed by the CHUV, the Swiss Federal Institute of Technology in Lausanne (EPFL) and the Swiss Vaccine Center as the common test (Fenwick et al. 2020). Reasons for this choice were the trimeric (entire S) target, the strong signal detected in persons with and without symptoms at the time of infection, the availability of IgG and IgA, the specificity of 99.7% and no cross-reactive antibodies in sera from people infected with pre-pandemic coronaviruses, a high sensitivity of 96.6% post 15 days of infection, superior performance over commercial tests in population-based samples that missed up to 45% of seropositive persons, the possibility for quantification, the availability of a neutralizing antibody test, the evidence of superior performance in population-based samples in comparison with six commercial tests, the availability of test material, and the possibility of use of this Luminex-based test in other laboratories in Switzerland (Fenwick et al. 2020).

### Longitudinal follow-up

A digital follow-up will be conducted for a duration of 6–12 months, depending on the progression of the pandemic, allowing for tracking the health status of individuals and for identifying new infections through reports of flu-like symptoms. The digital follow-up consists of weekly, brief status updates with respect to self-reported symptoms and risk exposures as well as monthly follow-ups. Details on the weekly and monthly follow-ups are found in Table 3, and the questionnaires are in the supplementary material. In some centers, groups of participants will further be invited to participate in repeated in-person visits for testing of their SARS-CoV-2 antibody levels.

### Statistical analysis plan and sample size calculation

There will be a centralized data management plan and a centralized process for analyses done on pooled data from all study sites. The primary endpoint for the overall study is the seroprevalence of SARS-CoV-2 antibodies in the general population at repeated time points during the pandemic in Switzerland. From the data collected within the seroprevalence studies of the randomly selected population sample, we will determine the age-specific and time-specific attack rate based on seroprevalence. We will also calculate the age-specific and region-specific cumulative incidence of seropositive individuals who have been asymptomatic, age-specific distribution of disease severity in seropositives who have been symptomatic, age-specific and disease severity-specific geometric mean level of IgG/IgA antibody titers, and lastly population groups most at risk of being seropositive (e.g., age groups, sex, occupation). To estimate seroprevalence, we use a Bayesian logistic regression model, accounting for the age and sex of the general population, and weighted for the sampling strategy (Stringhini et al. 2020). We integrate this regression model with a binomial model of the antibody test sensitivity and specificity to adjust the estimates for test performance while propagating uncertainty around test performance into final seroprevalence estimates. Recent observations suggest that antibodies could decrease if not disappear with time (Ibarrondo et al. 2020; Long et al. 2020). If confirmed in larger and prospectively planned studies (see Table 2), we will account for this trend by modeling the impact on the estimated proportion of participants having been infected.

The association of seroprevalence with participants’ characteristics, potential risk factors, and preventative measures will be assessed by mixed multiple linear and logistic regression analyses, with random effects for cantons, adjusted for potential confounders and will include interaction terms if reasonable. Analyses will be conducted in R (R Core Team 2020), with some sites conducting local analyses using R or other software (e.g., STATA).

The sample size provides enough statistical power to estimate the seroprevalence of SARS-CoV-2 antibodies with reasonable accuracy in each Canton, for different age groups, and at different points in time (sample sizes range from *n* = 1200–8640 per Canton; Table 1). Estimates are considered precise enough for informing policy-makers if the entire 90% credible interval leads to the same interpretation of seroprevalence and possible decisions. For specific groups of interest (e.g., age group 65+ or any subpopulation), and considering a serological test with 98% sensitivity and 99% specificity, a minimum population of 200 persons with an observed seroprevalence of 5% will yield 90% credible intervals of ± 2.5% for a posterior prevalence of 4.6%, of ± 3.5% for a posterior seroprevalence of 9.7% (observed prevalence 10%) and of ± 4.6% for a posterior seroprevalence of 19.9% (observed prevalence 20%). We consider these precisions enough for informing policy-makers. Site are free to have larger samples per group of interest, and we will adapt the sample size calculations once the seroprevalence is higher than 20%. For each age group, three sampling reserves (each representing 25% of the needed sample size) will additionally be drawn by the FSO. The reserves will be used in case of lower participation rate if the needed sample size is not attained.

## Results

The University of Geneva provided phase one estimates for Geneva by enrolling 2766 participants from 1399 households with a demographic difference that mirrored the Canton. When they began in April 2020, the seroprevalence was estimated at 4.8% (95% CI 2.4–8.0, *n* = 341) (Stringhini et al. 2020). By the fifth week, the seroprevalence had risen to 10.8% (95% CI 8.2–13.9, *n* = 775). The age group with highest risk of being seropositive was found to be those aged 20–49 years.

The centers of Zurich, Lausanne, Basel City/Land, Ticino, and Fribourg began recruitment for the second phase of the study and have recruited 5973 participants. Of those recruited, 1187, 1560, and 1677 participants have completed the first, second, and third weeks of the digital follow-up, respectively. A total of 900 participants have completed the first monthly follow-up.

Given the results from phase one from Geneva and the number of diagnosed cases of SARS-CoV-2 up to August 2020 across the Swiss Cantons, we expect declining seroprevalence rates from Southern to Northern and from Western to Eastern Switzerland. Thus, for phase two we may expect seroprevalences between 2 and 15% across regions. For phases three (November 2020) and four (March 2021), this may change considerably because the pandemic now has substantially different numbers of new cases within German- and French-speaking cantons.

## Discussion

Corona Immunitas is a research program coordinated by SSPH+, conducting longitudinal, population-based seroprevalence studies covering a number of Swiss Cantons as well as several seroepidemiological studies in specific subpopulations. The population-based seroprevalence studies are conducted on population samples that are representative of the Cantonal populations, ensured by a random selection of residents in the Cantonal population registries. Studies on subpopulations cover various populations and are detailed in Table 2. Studies are mostly cross-sectional and a number of them include nested longitudinal components to help capture much more than serology estimates of the population.

Corona Immunitas addresses a number of limitations of the current evidence in seroprevalence studies. One goal is to conduct studies with low risk of bias and a sampling strategy for both general and subpopulations that reflect the target populations of interest as much as possible. We use an accurate serology test and consistent questionnaires across sites to give a clearer picture of the pandemic. The longitudinal component of the program will provide guidance as to the extent and duration of immunity, as well as the long-term impact of the pandemic and lockdown measures. Therefore, Corona Immunitas gives policy-makers useful information for public health decisions. Several investigators of Corona Immunitas are members of the National COVID-19 Science Task Force of Switzerland that directly advises the FOPH and Federal Council (https://ncs-tf.ch/de/). Others are closely linked to cantonal health authorities and stakeholders. On the cantonal level, Corona Immunitas works with the respective health authorities and aims to establish a science to policy collaboration with the federation of Cantonal health directors. Finally, the program will provide information about the impact of the pandemic and the effectiveness of protective measures to decision makers for those persons with particularly exposed occupations and vulnerable persons.

Despite these efforts, there is potential for several limitations. First, even though efforts were made to recruit representative samples of the population by inviting randomly selected residents from cantonal population registries, we expect a relatively low participation rate which may introduce selection bias. Some reasons why individuals might not participate include lack of time and motivation, the fact that the assessment period of the second phase coincided with the Swiss summer holiday, the fear of being infected, and test fatigue (Bobrovitz et al. 2020). Other risks of selection bias that could artificially increase the estimated seroprevalence include stronger motivation to participate if they are symptomatic, were in contact with a person who tested positive for COVID-19, or travelled to a severely affected area. Although the coordination is strong on conceptual aspects of the study, practical aspects differ between study sites. Study sites share a common protocol, but some differences may still exist. To mitigate the risk that these differences impact on the results, the consortium will regularly compare SOPs and visit sites. Finally, antibodies may not be detectable in those who had a SARS-CoV-2 infection but with no or mild symptoms (Long et al. 2020) and estimates may therefore underestimate seroprevalence. However, the repeated serological testing will provide important insights into the potential disappearance of antibodies, factors associated with it and allow for correcting estimates of the proportion of people who had the infection.

One advantage of Corona Immunitas is to learn from other existing seroprevalence studies worldwide and the emergence of further evidence on the accuracy of serological tests as well as the nature of different tests. The use of a combined data management plan and nationwide serology test will guarantee interoperability and comparability. Additionally, the common work allows the consortium to cover different phases of the pandemic (https://www.corona-immunitas.ch/program) and to study subpopulations in a complementary way, which avoids redundancies and increases exhaustiveness.

Another advantage of the Corona Immunitas program is the combined use of serological testing and questionnaires. Self-reported data on socioeconomic characteristics, symptoms, and contact tracing will give rise to future analyses, which will further the understanding of the pandemic in more depth than serological testing alone. It will provide the possibility to assess severity of illness per infection more accurately, as well as transmission dynamics, and effect of socioeconomic characteristics. Transmission parameters are important for assessing transmission risks and shaping sanitary recommendations tailored to workspaces, schools, or the private sphere, e.g., in households or for families, especially those with elderly parents in nursing homes (McMichael et al. 2020). Socioeconomic characteristics also need to be addressed, especially social determinants of health (Khalatbari-Soltani et al. 2020) and their relation with the disproportionate impact of death and severe illness on social minorities (Saini 2020). These populations can be disproportionately affected by negative consequences of the pandemic from a physical health, mental health and a socioeconomic perspective. Additionally, the subsequent digital follow-up will provide data to help nourish a deeper understanding of the pandemic and its related sanitary measures on mental and physical health, and overall well-being of the population and society.

The Corona Immunitas program will inform on the prevalence of the population infected by region and its longitudinal components will inform on potential post-infection acquired immunity and its duration. Corona Immunitas is a unique nationwide research program that is centrally coordinated, which maintains the independence of all centers involved, ensuring both interoperability and comparability, and the adaptation of study designs to local needs. The Corona Immunitas consortium will generate reliable, comparable and high-quality data with extensive coverage of the Swiss geography and of several subpopulations of interest, to inform governmental and sector-specific decision making on the management of the SARS-CoV-2 pandemic. It can serve as a template for other regions and countries as it is important to have comparable data to fight this pandemic most efficiently.

## Electronic supplementary material

Below is the link to the electronic supplementary material.

Supplementary file1 (DOCX 127 kb)
