# Supplementary material for: Corona Immunitas: study protocol of a nationwide program of SARS-CoV-2 seroprevalence and seroepidemiologic studies in Switzerland
Source: Int J Public Health. 2020 Oct 24;65(9):1529–48. doi: 10.1007/s00038-020-01494-0 (PMC7584867; doi:10.1007/s00038-020-01494-0)
Supplement: Supplementary file 1 — Supplementary file1 (DOCX 127 kb) [file 38_2020_1494_MOESM1_ESM.docx]

**Supplementary material**

**Corona Immunitas: study protocol of a nationwide program of SARS-CoV-2 seroprevalence and seroepidemiologic studies in Switzerland**

International Journal of Public Health

| BASELINE QUESTIONNAIRE | |
| --- | --- |
|  |  |
| ***Email address*** *(if available)* | ____________________________________________ |
|  |  |
| **Part 1: Personal Details**  We will use this information to contact you (if you have given your consent) and to investigate whether there has been an accumulation of corona infections in certain areas. | |
| ***1.1 Date*** *(today’s)* | ____________________________________________ |
| ***1.2 Surname*** | ____________________________________________ |
| ***1.3 First Name*** | ____________________________________________ |
| ***1.4 Gender*** | - Male - Female - Other (please specify):   _________________________________ |
| ***1.5 Birthday*** | _____________________________________________ |
| ***1.6 Age*** | _____________________________________________ |

|  | | |
| --- | --- | --- |
| ***1.7 Home Address*** | | |
|  | 1.7.1 Street | ____________________________________________ |
|  | 1.7.2 House Number | ____________________________________________ |
|  | 1.7.3 ZIP/Postal Code | ____________________________________________ |
|  | 1.7.4 Location | ____________________________________________ |
|  | 1.7.5 Telephone Number 1 (Landline or Mobile) | ____________________________________________ |
|  | 1.7.6 Telephone Number 2 (Landline or Mobile) | ____________________________________________ |
|  | 1.7.7 AHV – Number (if known) | ____________________________________________ |
|  | |  |

| Record ID (not to be filled out) | | | | | ___________________________________________ | |
| --- | --- | --- | --- | --- | --- | --- |
|  | | | | | | |
| **Part 2: Information on your state of health and whether you belong to the group of particularly vulnerable persons.**  This part includes questions about whether you belong to the group of people who are particularly at risk of coronavirus infection (people with certain chronic diseases or overweight), questions about possible pregnancy, vaccinations and your blood type. | | | | | | |
| - 1. ***Do you suffer from one or more of the following diseases?*** *🡪 Please select yes or no for each disease.* | | | | | | |
|  | | | | | **Yes** | **No** |
| Cancer | | | | | O | O |
| Diabetes | | | | | O | O |
| Diseases and/or treatments that weaken the immune system | | | | | O | O |
| High blood pressure diagnosed by a doctor | | | | | O | O |
| Cardiovascular diseases (e.g. angina pectoris, peripheral vascular diseases, peripheral arterial occlusive disease, heart attack, stroke, heart failure) | | | | | O | O |
| Chronic respiratory diseases (e.g. chronic obstructive pulmonary disease (COPD), chronic bronchitis, emphysema, asthma) | | | | | O | O |
| Pollen allergy or hay fever | | | | | O | O |
| Other diseases | | | | | O | O |
|  | ***If other illnesses****, please specify:* | | | | ____________________________________________ | |
|  | | | | | | |
| ***2.2 What is your height?*** *(cm)* | | | | | ____________________________________________ | |
| ***2.3 What is your weight?*** *(kg)* | | | | | ____________________________________________ | |
| ***2.4 Are you pregnant?*** | | | | | O Yes | O No |
| ***2.5 What is your blood type?*** | | | | | - A - B - AB - 0 - I don’t know | |
| ***2.6 Have you ever been vaccinated against seasonal flu*** *(Influenza)****?*** | | | | | - Yes - No - I don’t know | |
|  | ***2.6.1 If yes****, in which year were you last vaccinated? (approximate year)* | | | | ____________________________________________ | |
| ***2.7 Have you ever been vaccinated against tuberculosis*** *(BCG)****?*** | | | | | - Yes - No - I don’t know | |
|  | | | | | | |
| **Part 3: Questions about possibly coronavirus symptoms and episodes, hospitalizations and tests carried out on you and others in your personal environment.** | | | | | | |
| - 1. ***Since January 2020, have you suffered from one or more of the following symptoms (for at least three days) that appeared unexpectedly and are not related to a possible chronic disease or an already known allergy?***   *🡪 Please select yes or no for each symptom.* | | | | | | |
|  | | | | | **Yes** | **No** |
| Feeling feverish | | | | | O | O |
| Body temperature of 38°C/100.4°F or more | | | | | O | O |
| Dry cough | | | | | O | O |
| Coughing with sputum | | | | | O | O |
| Bloody sputum | | | | | O | O |
| Runny or blocked nose | | | | | O | O |
| Sneezing | | | | | O | O |
| Sore throat | | | | | O | O |
| Shortness of breath | | | | | O | O |
| Breathing difficulties | | | | | O | O |
|  | | | | | Yes | **No** |
| Headaches | | | | | O | O |
| Muscle and/or limb pain | | | | | O | O |
| Pain in the chest, thorax and/or sternum | | | | | O | O |
| Tiredness or exhaustion | | | | | O | O |
| Loss of appetite | | | | | O | O |
| Nausea and/or vomiting | | | | | O | O |
| Diarrhea | | | | | O | O |
| Abdominal pain | | | | | O | O |
| Loss of smell and/or taste | | | | | O | O |
| Irritated and/or watery eyes | | | | | O | O |
| Other symptoms | | | | | O | O |
|  | ***If other symptoms****, please specify:* | | | | ____________________________________________ | |
| ***🡪 If all of the above are NO in section 3.1, please go directly to 3.2. If YES for one or more symptoms, please go to 3.1.1.*** | | | | | | |
|  | ***3.1.1 If you suffered from unexpected symptoms since January 2020:*** | | | | | |
|  | During how many **individual episodes** did these symptoms occur? (An episode means that you have had the symptoms for at least three days in a row) | | | | - During a **single** episode - During **two** different episodes - During **three or more** different episodes | |
|  | ***3.1.2 For each episode, please indicate when the new symptoms first appeared:*** | | | | | |
| • | **If an episode** occurred:  Episode 1. When did these new symptoms start and how long did they last? | | | | | |
|  | Start (approximate date) | | | | ____________________________________________ | |
|  | Number of days (approximately) | | | | ____________________________________________ | |
| • | **If two episodes** occurred:  Episode 2. When did these new symptoms start and how long did they last? | | | | | |
|  | Start (approximate date) | | | | ____________________________________________ | |
|  | Number of days (approximately) | | | | ____________________________________________ | |
| • | **If three or more** **episodes** occurred:  Episode 3. When did these new symptoms start and how long did they last? | | | | | |
|  | Start (approximate date) | | | | ____________________________________________ | |
|  | Number of days (approximately) | | | | ____________________________________________ | |
| ***If you believe that the reasons for these symptoms were not related to the coronavirus, you can describe it here:***  ____________________________________________________________________________________  ____________________________________________________________________________________ | | | | | | |
|  | ***3.1.3 Have you taken one or more medications during the last episode to relieve these symptoms?*** *(Multiple answers possible)* | | | | - No medication - Paracetamol (e.g. Dafalgan, NeoCitran, Pretuval, Panadol, Ben-U-RON, Zolben, etc.) - Cortisone, Prednisone - Non-steroidal analgesics/anti-inflammatory drugs (e.g. Ibuprofen, Irfen, Algifor, Brufen, etc.) - Other painkillers/inflammation inhibitors (e.g. Aspirin, Naproxen, Diclofenac, etc.) - Antiviral agents (e.g. Tamiflu, Ritonavir-Lopinavir, Remdesivir, etc.) - Antibiotics (e.g. Amoxicillin, Azithromycin, Bactrim, etc.) - Nasal sprays for asthma or chronic bronchitis (e.g. Atrovent, Bricanyl, Dospir, Seretide, Ventolin, Symbicort, Spiriva, etc.) - Hydroxychloroquin (Plaquentil) - Other medicines (please specify):   ________________________________ | |
|  |  | **If you have taken any medication**, were you recommended these drugs by a healthcare professional? | | | - Yes, by a doctor - Yes, by a pharmacist - No, by other persons (relatives, friends, etc.) or you have decided yourself | |
|  |  | **If you have taken any medication**, did you have to be treated in the hospital for the symptoms mentioned above? | | | O Yes | O No |
|  |  |  | ***If yes****, how long was this hospital stay?* | | | |
|  |  |  |  | Start (approximate date) | ____________________________________________ | |
|  |  |  |  | Number of days (approximately) | ____________________________________________ | |
|  | | | | | | |
| ***3.2 Have you ever been tested for the coronavirus*** *(SARS-CoV-2)* ***with a nasal or throat swab since January 2020?*** | | | | | - Yes - No - I don’t know / I don’t want to answer this question | |
|  | **3.2.1 If yes**, when exactly? (dd/mm/yyyy) | | | | ____________________________________________ | |
|  | **3.2.2 If yes**, the test result was | | | | - Positive (Virus present) - Negative (Virus not present) - Test result still pending - I don’t know / I don’t want to answer this question | |
| ***3.3 Have you ever had a blood test*** *(Serology)* ***to detect antibodies against the coronavirus*** *(SARS-CoV-2)* ***since January 2020?*** | | | | | - Yes - No - I don’t know / I don’t want to answer this question | |
|  | **3.3.1 If yes**, when exactly? (dd/mm/yyyy) | | | | ____________________________________________ | |
|  | **3.3.2 If yes**, the test result was | | | | - Positive (Antibodies present) - Negative (Antibodies not present) - Test result still pending   I don’t know / I don’t want to answer this question | |
|  | | | | | | |
| ***3.4 How many people in your immediate environment*** *(who either live in the same household or with whom you are in regular close contact)* ***have developed symptoms that indicate a COVID-19 disease*** *(fever, cough, tiredness, shortness of breath, muscle aches, etc.)****, without being tested?*** *(Please provide a number)*  ***🡪*** *If 0, please go directly to* ***3.5.*** | | | | | | ______________________ |

|  | ***3.4.1 If more than 0*** *(zero),* *please indicate for each person their age, gender, whether they live in the same household and when their symptoms occurred (if more than two people, please provide the same information at the end of the questionnaire)* | | |
| --- | --- | --- | --- |
|  | • | **If** one person: Person 1 |  |
|  |  | Age | ____________________________________________ |
|  |  | Gender | - Male - Female |
|  |  | Person lives in the same household | - Yes - No |
|  |  | *When did these new symptoms start and approximately how long did they last?* | |
|  |  | Start (approximate date) | ____________________________________________ |
|  |  | Number of days (approximately) | ____________________________________________ |
|  | • | **If** two people: Person 2 |  |
|  |  | Age | ____________________________________________ |
|  |  | Gender | - Male - Female |
|  |  | Person lives in the same household | - Yes - No |
|  |  | *When did these new symptoms start and approximately how long did they last?* | |
|  |  | Start (approximate date) | ____________________________________________ |
|  |  | Number of days (approximately) | ____________________________________________ |

|  | | | | | | | | |
| --- | --- | --- | --- | --- | --- | --- | --- | --- |
| ***3.5 How many people in your immediate vicinity*** *(who either live in the same household or with whom you have regular close contact) tested positive for the coronavirus* *(SARS-CoV2)****?*** *(please provide a number)*  *🡪 If 0, please go directly to* ***4.*** | | | | | | ______________________ | | |
|  | ***If more than 0*** *(zero)****,*** *for each person who tested positive, include their date of test, age, gender and whether they live in the same household as you (If more than two people, please provide the same information about these people at the end of the questionnaire)* | | | | | | | |
|  | • | **If** one person: Person 1 | |  | | | | |
|  |  | Date (approximate) | | ____________________________________________ | | | | |
|  |  | Age | | ____________________________________________ | | | | |
|  |  | Gender | | - Male - Female | | | | |
|  |  | Person lives in the same household | | - Yes - No | | | | |
|  | • | **If** two people: Person 2 | |  | | | | |
|  |  | Date (approximate) | | ____________________________________________ | | | | |
|  |  | Age | | ____________________________________________ | | | | |
|  |  | Gender | | - Male - Female | | | | |
|  |  | Person lives in the same household | | - Yes - No | | | | |
|  | | | | | | | | |
| **Part 4: Sociodemographic data, questions about work and household.** | | | | | | | | |
| ***4.1 Do you smoke cigarettes?*** | | | | - Yes, I smoke daily - Yes, I smoke occasionally - No, but in the past I have smoked daily (ex-smoker) (Please go directly to **4.1.3.**) - No, never or less than 100 cigarettes in my life (Please go directly to **4.2.**) | | | | |
|  | **4.1.1 If daily**, how many cigarettes per day do you smoke on average? | | | ____________________________________________ | | | | |
|  | **4.1.2 If occasionally**, how many cigarettes per month do you smoke on average? | | | ____________________________________________ | | | | |
|  | **4.1.3 If you smoked every day in the past,** when did you stop smoking? (mm/yyyy) | | | ____________________________________________ | | | | |
| ***4.2 Do you currently use tobacco products other than cigarettes*** *(e.g. cigars, pipes, IQOS, Glo, Ploom, shisha, etc.)****?*** | | | | - Yes, everyday - Yes, occasionally - No, not currently or only in the past (Please go directly to **4.3.**) | | | | |
|  | **4.2.1 If yes**, which product(s)? | | | ____________________________________________ | | | | |
| ***4.3 Do you currently use electronic cigarettes*** *(Vaping)****?*** | | | | - Yes - No (Please go directly to **4.4.**) | | | | |
|  | **4.3.1 If yes**, what concentration of nicotine do you use (mg/ml)? | | | - 0 (no nicotine) - Up to 6 - More than 6 and less than 13 - 13 or more | | | | |
|  | | | | | | | | |
| ***4.4 What is your nationality*** *(multiple answers possible)* | | | | - Switzerland - Germany - France - Italy - Spain - Portugal - Turkey - Serbia - Montenegro - Kosovo - Croatia - Austria - Great Britain - USA - Other (please specify):   _________________________________ | | | | |
| ***4.5 What is your mother tongue?*** *(multiple answers possible)* | | | | - Swiss-german - German - French - Italian - Spanish - Portuguese - Turkish - Serbian - Albanian - Montenegrin (Crnogorski) - Croatian - Bosnian - English - Other (please specify):   _________________________________ | | | | |
|  | | | | | | | | |
| ***4.6 How many people currently live in the same household as you?*** *(please enter a number)*  *🡪 If 0, please go directly to* ***4.7.*** | | | | | | ______________________ | | |
|  | **4.6.1 If several people** live in your household, please indicate the age and gender of each person (if more than 4 other people live in your household, please provide the same information for these people at the end of the questionnaire) | | | | | | | |
|  | • | If one other person: Person 1 | |  | | | | |
|  |  | Age | | _______________________________________________ | | | | |
|  |  | Gender | | O Male | | | O Female | |
|  | • | If two other people: Person 2 | |  | | | | |
|  |  | Age | | _______________________________________________ | | | | |
|  |  | Gender | | O Male | | | Gender | |
|  | • | If three other people: Person 3 | |  | | | | |
|  |  | Age | | _______________________________________________ | | | | |
|  |  | Gender | | O Male | | | Gender | |
|  | • | If four other people: Person 4 | |  | | | | |
|  |  | Age | | _______________________________________________ | | | | |
|  |  | Gender | | O Male | | | Gender | |
|  | | | | | | | | |
| ***4.7 Right now you are*** *(multiple answers possible)* | | | | - Retired (Please go to **4.8.**) - In training/at university (majority)   (please go to **4.7.3.**)   - Self-employed (please go to **4.7.1.**) - Employed (please go to **4.7.1.**) - Not working (please go to **4.8.**) - Other (please specify):   _______________________________ | | | | |
|  | **4.7.2 If you are employed**, how did you travel to your place of work before the coronavirus epidemic (majority)? | | | - I live in my workplace - On foot or by bike - By public transportation - By car | | | | |
|  | **4.7.3 If you are employed or in training/studying**, have your working conditions or the scope of your work changed since the outbreak of the coronavirus? | | | - Yes - No (please go to **4.8.**) | | | | |
|  |  | **If yes,** please specify | | **Yes, temporarily during the lockdown** | **Yes, permanently** | | | **No** |
|  |  | Changes in the scope of work | | O | O | | | O |
|  |  |  | Decreased by (%) | ____________________________________________ | | | | |
|  |  |  | Increased by (%) | ____________________________________________ | | | | |
|  |  | I work in home office (fully or partially) | | O | O | | | O |
|  |  | I am on sick leave (in whole or in part) | | O | O | | | O |
|  |  | I have suspended my professional activities because I am one of the people most at risk from the virus and because I can do my job at home | | O | O | | | O |
|  |  |  | | **Yes, temporarily during the lockdown** | **Yes, permanently** | | | **No** |
|  |  | I lost my job | | O | O | | | O |
|  |  | I am self-employed and have lost jobs | | O | O | | | O |
|  |  | **If others**, please specify: | | ____________________________________________ | | | | |
|  |  | **If** there has been a change in **your work situation** due to the coronavirus epidemic, will you find yourself in a **financially difficult situation** as a result? | | - Yes, very - Yes, a little - Not really - No - I don’t know | | | | |
|  | | | | | | | | |
| ***4.8 What is the highest education you have completed*** *(with certificate/diploma)****?*** | | | | - No school certificate - Mandatory school - Professional training (including diploma) - Matura, Abitur, vocational baccalaureate - Higher technical college (PH, etc.) or university of applied sciences (FH, etc.) - University studies (including polytechnic) | | | | |
| ***4.9 What is your current monthly*** *(gross)* ***household income?*** | | | | ____________________________________________ | | | | |
| ***4.10 What is your current monthly*** *(gross)* ***household income?*** | | | | - < CHF 3’000 - CHF 3'000 – 6’000 - CHF 6'000 – 9’000 - CHF 9'000 – 12’000 - CHF 12'000 – 15’000 - CHF 15’000- 18’000 - CHF 18'000 – 21’000 - > CHF 21’000 | | | | |

|  | | | | | | | |
| --- | --- | --- | --- | --- | --- | --- | --- |
| **Part 5: Questions about compliance with the measures, contact with others and your well-being.** | | | | | | | |
| ***5.1 In the last seven days, how many times have you …*** | | | | | | | |
|  | | **Never** | | **Very rare** | **Occasionally** | **Frequently** | **Always** |
| … implemented the recommended measures for “**social distancing”** (no handshaking or hugging, keep the distance of 2 meters, etc.)? | | O | | O | O | O | O |
| … implemented the recommendation to **stay at home** (stay at home whenever possible, avoid unnecessary activities outside the home, etc.)? | | O | | O | O | O | O |
| … worn a **mask** to protect yourself and others from the coronavirus (SARS-CoV-2)? | | O | | O | O | O | O |
| … worn **gloves** in public (when shopping in the supermarket, on public transporation, etc.), to protect yourself and others from the coronavirus? | | O | | O | O | O | O |
| … implemented the recommended **hygiene measures** (washing hands regularly, sneezing into the elbow, using tissues, etc.)? | | O | | O | O | O | O |
| ***5.2 On average, how many times a week do you or other people you live with go shopping?*** *(please enter a number)* | | | | | | | |
| **5.2.1** Before the lockdown (until about mid-March) | | | ____________________________________________ | | | | |
| **5.2.2** During the lockdown (mid-March until about mid-May) | | | ____________________________________________ | | | | |
| **5.2.3** Now | | | ____________________________________________ | | | | |
| ***5.3 How many people have you met on average per week (more than 15 minutes and less than 2 meters distance) apart from the people you live with?*** *(Please give a number)* | | | | | | | |
| **5.3.1** Before the lockdown (until about mid-March) | | | ____________________________________________ | | | | |
| **5.3.2** During the lockdown (mid-March until about mid-May) 🡪If more than 0, please go to **5.3.4**. | | | ____________________________________________ | | | | |
| **5.3.3** Now | | | ____________________________________________ | | | | |
| **5.3.4** In what context did you meet these people during the lockdown? | | | - Meeting with friends or family members - Work - Travel (public transport) - Sports - Other (please specify):   _________________________________ | | | | |
| ***5.4 How often have you traveled abroad since the end of January 2020*** *(apart from professional activities in the border region)****?***  *🡪 If 0 please go to* ***5.5****.* | | | ____________________________________________ | | | | |
| • | **If once**: Trip 1 (the last) | |  | | | | |
|  | To (country) | | ____________________________________________ | | | | |
|  | Departure date (dd/mm/yyyy) | | ____________________________________________ | | | | |
|  | Return date (dd/mm/yyyy) | | ____________________________________________ | | | | |
| • | **If twice**: Trip 2 (the second to last) | |  | | | | |
|  | To (country) | | ____________________________________________ | | | | |
|  | Departure date (dd/mm/yyyy) | | ____________________________________________ | | | | |
|  | Return date (dd/mm/yyyy) | | ____________________________________________ | | | | |
|  | | | | | | | |

|  | | | | | |
| --- | --- | --- | --- | --- | --- |
| ***5.5 How worried are you about the current coronavirus situation in the following areas:*** | | | | | |
|  | **Not at all** | **A little** | **Moderate** | **Very** | **Extreme** |
| The consequences for my health | O | O | O | O | O |
| The health of relatives and friends | O | O | O | O | O |
| The risk of exposing myself to the virus | O | O | O | O | O |
| The risk of spreading the virus | O | O | O | O | O |
| My own economic and/or professional situation | O | O | O | O | O |
| The economic and/or professional situation of relatives and friends | O | O | O | O | O |
| The general economic situation in Switzerland | O | O | O | O | O |
| The quality of my family relationships | O | O | O | O | O |
| The quality of my personal relationships (e.g. with friends and colleagues) | O | O | O | O | O |
| The freedom of Swiss citizens | O | O | O | O | O |
| The privacy of Swiss citizens | O | O | O | O | O |

| WEEKLY FOLLOW-UP | | | | | | | | | |
| --- | --- | --- | --- | --- | --- | --- | --- | --- | --- |
|  | | | | |  | | | | |
| ***1 Date*** *(today’s)* | | | | | ____________________________________________ | | | | |
|  | | | | | | | | |  |
|  | |  | | | |  |  |  |  |
| ***2.1 Over the past 7 days, have you had any new symptoms unrelated to pre-existing chronic illness or allergies?*** | | | O Yes | | | | O No |  |  |
| *🡪 If NO, please go directly to 3.1****.*** | | |  | | | |  |  |  |
| - 1. ***During the last 7 days, have you had the following symptoms (new symptoms unrelated to pre-existing chronic illness or allergies?***   *🡪 Please select yes or no for each symptom.* | | | | | | | |  |  |
|  | | | **Yes** | | | | **No** |  |  |
| Feeling feverish | | | O | | | | O |  |  |
| Body temperature of 38°C/100.4°F or more | | | O | | | | O |  |  |
| Dry cough | | | O | | | | O |  |  |
| Coughing with sputum | | | O | | | | O |  |  |
| Bloody sputum | | | O | | | | O |  |  |
| Runny or blocked nose | | | O | | | | O |  |  |
| Sneezing | | | O | | | | O |  |  |
| Sore throat | | | O | | | | O |  |  |
| Shortness of breath | | | O | | | | O |  |  |
| Breathing difficulties | | | O | | | | O |  |  |
| Headaches | | | O | | | | O |  |  |
| Muscle and/or limb pain | | | O | | | | O |  |  |
| Pain in the chest, thorax and/or sternum | | | O | | | | O |  |  |
|  | | | **Yes** | | | | **No** |  |  |
| Tiredness or exhaustion | | | O | | | | O |  |  |
| Loss of appetite | | | O | | | | O |  |  |
| Nausea and/or vomiting | | | O | | | | O |  |  |
| Diarrhea | | | O | | | | O |  |  |
| Abdominal pain | | | O | | | | O |  |  |
| Loss of smell and/or taste | | | O | | | | O |  |  |
| Irritated and/or watery eyes | | | O | | | | O |  |  |
| Other symptoms | | | O | | | | O |  |  |
|  | ***If other symptoms****, please specify:* | | ____________________________________________ | | | | |  |  |
|  | | | |  | | | | |  |

| ***3.1 Have you taken one or more medications during the last episode to relieve these symptoms?*** *(Multiple answers possible)* | | | | | | - No medication - Paracetamol (e.g. Dafalgan, NeoCitran, Pretuval, Panadol, Ben-U-RON, Zolben, etc.) - Cortisone, Prednisone - Non-steroidal analgesics/anti-inflammatory drugs (e.g. Ibuprofen, Irfen, Algifor, Brufen, etc.) - Other painkillers/inflammation inhibitors (e.g. Aspirin, Naproxen, Diclofenac, etc.) - Antiviral agents (e.g. Tamiflu, Ritonavir-Lopinavir, Remdesivir, etc.) - Antibiotics (e.g. Amoxicillin, Azithromycin, Bactrim, etc.) - Nasal sprays for asthma or chronic bronchitis (e.g. Atrovent, Bricanyl, Dospir, Seretide, Ventolin, Symbicort, Spiriva, etc.) - Hydroxychloroquin (Plaquentil) - Other medicines (please specify):   ________________________________ | | | | | | | | |  |  |  |
| --- | --- | --- | --- | --- | --- | --- | --- | --- | --- | --- | --- | --- | --- | --- | --- | --- | --- |
|  | **If you have taken any medication**, were you recommended these drugs by a healthcare professional? | | | | | - Yes, by a doctor - Yes, by a pharmacist - No, by other persons (relatives, friends, etc.) or you have decided yourself | | | | | | | | |  |  |  |
|  | **If you have taken any medication**, did you have to be treated in the hospital for the symptoms mentioned above? | | | | | O Yes | | | | | | O No | | |  |  |  |
|  |  | | ***If yes****, how long was this hospital stay?* | | | | | | | | | | | |  |  |  |
|  |  | |  | Start (approximate date) | | ____________________________________________ | | | | | | | | |  |  |  |
|  |  | |  | Number of days (approximately) | | ____________________________________________ | | | | | | | | |  |  |  |
|  | | | | | | | | | | | | | | | | |  |
| ***3.2 Have you ever been tested for the coronavirus*** *(SARS-CoV-2)* ***with a nasal or throat swab since January 2020?*** | | | | | | | | - Yes - No - I don’t know / I don’t want to answer this question | | | | | | | | |  |
| **3.2.1 If yes**, when exactly? (dd/mm/yyyy) | | | | | | ____________________________________________ | | | | | | | | |  |  |  |
| **3.2.2 If yes**, the test result was | | | | | | - Positive (Virus present) - Negative (Virus not present) - Test result still pending - I don’t know / I don’t want to answer this question | | | | | | | | |  |  |  |
| ***3.3 Have you ever had a blood test*** *(Serology)* ***to detect antibodies against the coronavirus*** *(SARS-CoV-2)* ***since January 2020?*** | | | | | | | | - Yes - No - I don’t know / I don’t want to answer this question | | | | | | | | |  |
| **3.3.1 If yes**, when exactly? (dd/mm/yyyy) | | | | | | ____________________________________________ | | | | | | | | |  |  |  |
| **3.3.2 If yes**, the test result was | | | | | | - Positive (Antibodies present) - Negative (Antibodies not present) - Test result still pending   I don’t know / I don’t want to answer this question | | | | | | | | |  |  |  |
|  | | | | | | | | | | | | | | | | |  |
|  | | | | | | | | | | | | | | | | |  |
| ***4.1 In the last seven days, how many times have you …*** | | | | | | | | | | | | | | | | |  |
|  | | | | | **Never** | | | | **Very rare** | | **Occasionally** | | **Frequently** | **Always** | | |  |
| … implemented the recommended measures for “**social distancing”** (no handshaking or hugging, keep the distance of 2 meters, etc.)? | | | | | O | | | | O | | O | | O | O | | |  |
| … implemented the recommendation to **stay at home** (stay at home whenever possible, avoid unnecessary activities outside the home, etc.)? | | | | | O | | | | O | | O | | O | O | | |  |
| … worn a **mask** to protect yourself and others from the coronavirus (SARS-CoV-2)? | | | | | O | | | | O | | O | | O | O | | |  |
| … implemented the recommended **hygiene measures** (washing hands regularly, sneezing into the elbow, using tissues, etc.)? | | | | | O | | | | O | | O | | O | O | | |  |
|  | | | | |  | | | |  | |  | |  |  | | |  |
|  | | | | | **Not at all** | | | |  | |  | |  | **Very high** | | |  |
| **4.2 In the last 7 days, do you think the risk of being infected with COVID-19 is…** *(Mark on the line where you believe the risk is; if you do not know, please check “I don’t know”)* | | | | |  | | | |  | |  | |  |  | | |  |
| - I don’t know | | | | | | | | | |  |  |  |  |  |  |  |  |
| ***4.3 For the past 7 days, have you been forced to stay at home because of exposure to coronavirus (SARS-CoV-2)?*** | | | | | | | | | | - Yes, because I have tested positive for coronavirus and/or have symptoms - Yes, because I was in contact with a person who tested positive for coronavirus - Yes, because a household member has tested positive for coronavirus or has symptoms - Yes, because I am at risk (because of my age or a previous illness - Yes, for another reason - No - I don’t know/don’t want to answer | | | | | | | |
|  | | ***If yes for another reason****, please specify:* | | | | | ____________________________________________ | | | | | | | | |  |  |
| ***4.4 If you stayed at home (or are still at home), who asked you to do so*** | | | | | | | | | | - I have followed the prescription of my doctor or the health specialist following me - I have followed the health recommendations currently applied in my canton - I have followed the recommendations of my employer or my occupational physician - I followed the recommendations of my relatives (friends, family) - I decided to stay home by myself - Other | | | | | | | |
|  | | ***If other****, please specify:* | | | | | ____________________________________________ | | | | | | | | |  |  |

| MONTHLY FOLLOW-UP | | | | | | | | | |
| --- | --- | --- | --- | --- | --- | --- | --- | --- | --- |
|  | | | | |  | | | | |
| ***1 Date*** *(today’s)* | | | | | ____________________________________________ | | | | |
|  | | | | | | | | |  |
|  | |  | | | |  |  |  |  |
| ***2.1 Over the past 7 days, have you had any new symptoms unrelated to pre-existing chronic illness or allergies?*** | | | O Yes | | | | O No |  |  |
| *🡪 If NO, please go directly to 3.1****.*** | | |  | | | |  |  |  |
| - 1. ***During the last 7 days, have you had the following symptoms (new symptoms unrelated to pre-existing chronic illness or allergies?***   *🡪 Please select yes or no for each symptom.* | | | | | | | |  |  |
|  | | | **Yes** | | | | **No** |  |  |
| Feeling feverish | | | O | | | | O |  |  |
| Body temperature of 38°C/100.4°F or more | | | O | | | | O |  |  |
| Dry cough | | | O | | | | O |  |  |
| Coughing with sputum | | | O | | | | O |  |  |
| Bloody sputum | | | O | | | | O |  |  |
| Runny or blocked nose | | | O | | | | O |  |  |
| Sneezing | | | O | | | | O |  |  |
| Sore throat | | | O | | | | O |  |  |
| Shortness of breath | | | O | | | | O |  |  |
| Breathing difficulties | | | O | | | | O |  |  |
| Headaches | | | O | | | | O |  |  |
| Muscle and/or limb pain | | | O | | | | O |  |  |
| Pain in the chest, thorax and/or sternum | | | O | | | | O |  |  |
|  | | | **Yes** | | | | **No** |  |  |
| Tiredness or exhaustion | | | O | | | | O |  |  |
| Loss of appetite | | | O | | | | O |  |  |
| Nausea and/or vomiting | | | O | | | | O |  |  |
| Diarrhea | | | O | | | | O |  |  |
| Abdominal pain | | | O | | | | O |  |  |
| Loss of smell and/or taste | | | O | | | | O |  |  |
| Irritated and/or watery eyes | | | O | | | | O |  |  |
| Other symptoms | | | O | | | | O |  |  |
|  | ***If other symptoms****, please specify:* | | ____________________________________________ | | | | |  |  |
|  | | | |  | | | | |  |

| ***3.1 Have you taken one or more medications during the last episode to relieve these symptoms?*** *(Multiple answers possible)* | | | | | | - No medication - Paracetamol (e.g. Dafalgan, NeoCitran, Pretuval, Panadol, Ben-U-RON, Zolben, etc.) - Cortisone, Prednisone - Non-steroidal analgesics/anti-inflammatory drugs (e.g. Ibuprofen, Irfen, Algifor, Brufen, etc.) - Other painkillers/inflammation inhibitors (e.g. Aspirin, Naproxen, Diclofenac, etc.) - Antiviral agents (e.g. Tamiflu, Ritonavir-Lopinavir, Remdesivir, etc.) - Antibiotics (e.g. Amoxicillin, Azithromycin, Bactrim, etc.) - Nasal sprays for asthma or chronic bronchitis (e.g. Atrovent, Bricanyl, Dospir, Seretide, Ventolin, Symbicort, Spiriva, etc.) - Hydroxychloroquin (Plaquentil) - Other medicines (please specify):   ________________________________ | | | | | | | | |  |  |  |
| --- | --- | --- | --- | --- | --- | --- | --- | --- | --- | --- | --- | --- | --- | --- | --- | --- | --- |
|  | **If you have taken any medication**, were you recommended these drugs by a healthcare professional? | | | | | - Yes, by a doctor - Yes, by a pharmacist - No, by other persons (relatives, friends, etc.) or you have decided yourself | | | | | | | | |  |  |  |
|  | **If you have taken any medication**, did you have to be treated in the hospital for the symptoms mentioned above? | | | | | O Yes | | | | | | O No | | |  |  |  |
|  |  | | ***If yes****, how long was this hospital stay?* | | | | | | | | | | | |  |  |  |
|  |  | |  | Start (approximate date) | | ____________________________________________ | | | | | | | | |  |  |  |
|  |  | |  | Number of days (approximately) | | ____________________________________________ | | | | | | | | |  |  |  |
|  | | | | | | | | | | | | | | | | |  |
| ***3.2 Have you ever been tested for the coronavirus*** *(SARS-CoV-2)* ***with a nasal or throat swab since January 2020?*** | | | | | | | | - Yes - No - I don’t know / I don’t want to answer this question | | | | | | | | |  |
| **3.2.1 If yes**, when exactly? (dd/mm/yyyy) | | | | | | ____________________________________________ | | | | | | | | |  |  |  |
| **3.2.2 If yes**, the test result was | | | | | | - Positive (Virus present) - Negative (Virus not present) - Test result still pending - I don’t know / I don’t want to answer this question | | | | | | | | |  |  |  |
| ***3.3 Have you ever had a blood test*** *(Serology)* ***to detect antibodies against the coronavirus*** *(SARS-CoV-2)* ***since January 2020?*** | | | | | | | | - Yes - No - I don’t know / I don’t want to answer this question | | | | | | | | |  |
| **3.3.1 If yes**, when exactly? (dd/mm/yyyy) | | | | | | ____________________________________________ | | | | | | | | |  |  |  |
| **3.3.2 If yes**, the test result was | | | | | | - Positive (Antibodies present) - Negative (Antibodies not present) - Test result still pending   I don’t know / I don’t want to answer this question | | | | | | | | |  |  |  |
|  | | | | | | | | | | | | | | | | |  |
|  | | | | | | | | | | | | | | | | |  |
| ***4.1 In the last seven days, how many times have you …*** | | | | | | | | | | | | | | | | |  |
|  | | | | | **Never** | | | | **Very rare** | | **Occasionally** | | **Frequently** | **Always** | | |  |
| … implemented the recommended measures for “**social distancing”** (no handshaking or hugging, keep the distance of 2 meters, etc.)? | | | | | O | | | | O | | O | | O | O | | |  |
| … implemented the recommendation to **stay at home** (stay at home whenever possible, avoid unnecessary activities outside the home, etc.)? | | | | | O | | | | O | | O | | O | O | | |  |
| … worn a **mask** to protect yourself and others from the coronavirus (SARS-CoV-2)? | | | | | O | | | | O | | O | | O | O | | |  |
| … implemented the recommended **hygiene measures** (washing hands regularly, sneezing into the elbow, using tissues, etc.)? | | | | | O | | | | O | | O | | O | O | | |  |
|  | | | | |  | | | |  | |  | |  |  | | |  |
|  | | | | | **Not at all** | | | |  | |  | |  | **Very high** | | |  |
| **4.2 In the last 7 days, do you think the risk of being infected with COVID-19 is…** *(Mark on the line where you believe the risk is; if you do not know, please check “I don’t know”)* | | | | |  | | | |  | |  | |  |  | | |  |
| - I don’t know | | | | | | | | | |  |  |  |  |  |  |  |  |
| ***4.3 For the past 7 days, have you been forced to stay at home because of exposure to coronavirus (SARS-CoV-2)?*** | | | | | | | | | | - Yes, because I have tested positive for coronavirus and/or have symptoms - Yes, because I was in contact with a person who tested positive for coronavirus - Yes, because a household member has tested positive for coronavirus or has symptoms - Yes, because I am at risk (because of my age or a previous illness - Yes, for another reason - No - I don’t know/don’t want to answer | | | | | | | |
|  | | ***If yes for another reason****, please specify:* | | | | | ____________________________________________ | | | | | | | | |  |  |

|  | | | | | | | | |
| --- | --- | --- | --- | --- | --- | --- | --- | --- |
| ***5 How worried are you about the current coronavirus situation in the following areas:*** | | | | | | | | |
|  | **Not at all** | | **A little** | | | **Moderate** | **Very** | **Extreme** |
| The consequences for my health | O | | O | | | O | O | O |
| The health of relatives and friends | O | | O | | | O | O | O |
| The risk of exposing myself to the virus | O | | O | | | O | O | O |
| The risk of spreading the virus | O | | O | | | O | O | O |
| My own economic and/or professional situation | O | | O | | | O | O | O |
| The economic and/or professional situation of relatives and friends | O | | O | | | O | O | O |
| The general economic situation in Switzerland | O | | O | | | O | O | O |
| The quality of my family relationships | O | | O | | | O | O | O |
| The quality of my personal relationships (e.g. with friends and colleagues) | O | | O | | | O | O | O |
| The freedom of Swiss citizens | O | | O | | | O | O | O |
| The privacy of Swiss citizens | O | | O | | | O | O | O |
|  | | | | | | | | |
| ***6 To which extent do you think the following people are worried about the current coronavirus (SARS-CoV-2) situation:*** | | | | | | | | |
|  | **Not at all** | | **A little** | | | **Moderate** | **Very** | **Extreme** |
| My family members | O | | O | | | O | O | O |
| Other people around me (e.g. friends, neighbors, colleagues) | O | | O | | | O | O | O |
| Public health authorities | O | | O | | | O | O | O |
| News media | O | | O | | | O | O | O |
| People on social media | O | | O | | | O | O | O |
| Healthcare practitioners | O | | O | | | O | O | O |
| Politicians | O | | O | | | O | O | O |
|  | | | | | | | | |
| ***7.1 During the past 7 days, how often did you feel that you lacked companionship****)****?*** | | | | - Never - Rarely - Occasionally - Most of the time - Always | | | | |
| ***7.2 During the past 7 days, how often did you feel left out?*** | | | | - Never - Rarely - Occasionally - Most of the time - Always | | | | |
| ***7.3 During the past 7 days, how often did you feel isolated from others?*** | | | | - Never - Rarely - Occasionally - Most of the time - Always | | | | |
|  | | | | | | | | |
| ***8.1 Please read each statement and indicate a number 0, 1, 2, or 3, which indicates how much the statement applied to you over the past week. There are no right or wrong answers. Do not spend too much time on any statement:*** | | | | | | | | |
|  |  | | **0 – Did not apply to me at all** | | | **1 – Applied to me to some degree, or some of the time** | **2 – Applied to me to a considerable degree, or a good part of the time** | **3 – Applied to me very much, or most of the time** |
| I found it hard to wind down | |  | O | | | O | O | O |
| I was aware of dryness of my mouth | |  | O | | | O | O | O |
| I couldn’t seem to experience any positive feelings at all | |  | O | | | O | O | O |
| I experienced breathing difficulty (e.g. excessively rapid breathing, breathlessness in the absence of physical exertion) | |  | O | | | O | O | O |
| I found it difficult to work up the initiative to do things | |  | O | | | O | O | O |
| I tended to over-react to situations | |  | O | | | O | O | O |
| I experienced trembling (e.g. in the hands) | |  | O | | | O | O | O |
| I felt that I was using a lot of nervous energy | |  | O | | | O | O | O |
| I was worried about situations in which I might panic and make a fool of myself | |  | O | | | O | O | O |
| I felt that I had nothing to look forward to | |  | O | | | O | O | O |
|  | | | | | | | | |
| ***8.2 Please read each statement and indicate a number 0, 1, 2, or 3, which indicates how much the statement applied to you over the past week. There are no right or wrong answers. Do not spend too much time on any statement:*** | | | | | | | | |
|  |  | | **0 – Did not apply to me at all** | | | **1 – Applied to me to some degree, or some of the time** | **2 – Applied to me to a considerable degree, or a good part of the time** | **3 – Applied to me very much, or most of the time** |
| I found myself getting agitated | |  | O | | | O | O | O |
| I found it difficult to relax | |  | O | | | O | O | O |
| I felt down-hearted and blue | |  | O | | | O | O | O |
| I was intolerant of anything that kept me from getting on with what I was doing | |  | O | | | O | O | O |
| I felt I was close to panic | |  | O | | | O | O | O |
| I was unable to become enthusiastic about anything | |  | O | | | O | O | O |
| I felt I wasn’t worth much as a person | |  | O | | | O | O | O |
| I felt that I was rather touchy | |  | O | | | O | O | O |
| I was aware of the action of my heart in the absence of physical exertion (e.g. sense of heart rate increase, heart missing a beat) | |  | O | | | O | O | O |
| I felt scared without any good reason | |  | O | | | O | O | O |
| I felt that life was meaningless | |  | O | | | O | O | O |
|  | | | | | | | | |
| ***9.1 Did you need medical treatment because of an ongoing illness?*** | | | | |  |  |  |  |
| ***9.2 If yes,*** *have you changed your ongoing medical treatment during the past month****?*** | | | | | - No, I have not changed my medical treatment (Please go directly to **9.3.**) - Yes, I have had problems obtaining my usual treatment (Please go directly to 9.4**.**) - Yes, I have stopped my treatment (for example: corticosteroids, anti-inflammatory drugs) so as not to risk aggravating a potential coronavirus infection (Please go directly to **9.5.**) - Yes, for another reason (Please go directly to **9.6.**) | | | |
| ***9.3 Have you been afraid of getting infected with the coronavirus (SARS-CoV-2) by going for treatment?*** | | | | | - Yes, very afraid - Yes, fairly afraid - No, not very afraid - No, not afraid of everything - Not concerned | | | |
| ***9.4 Why did you have problems obtaining your usual treatment*** *(select all that apply)****?*** | | | | | - My appointment was postponed or cancelled - The health professional had closed his/her office - I couldn’t have my children looked after to go for treatment - Other reason   *Please specify:* __________________________ | | | |
| ***9.5 Why have you stopped your treatment*** *(select all that apply)****?*** | | | | | - Because I was too afraid of being infected during treatment - Because I wanted to reduce my trips outside the home - Because I wanted to protect other members in my family from getting infected - Other reason - *Please specify:* __________________________ | | | |
| ***9.6 If yes for another reason****, please specify:* | | | | | ____________________________________________ | | | |
|  | | | | | | | | |
| ***The SwissCovid App has been launched by the Swiss Federal Office of Public Health to warn smartphone users in case of possible exposure risks. The app records if a contact has been in close proximity of 1.5m or less for longer than 15 minutes. If an app user tested positive for the coronavirus, she or he can anonymously notify other app users who were in close proximity during the infectious period.*** | | | | | | | | |
| ***10.1 Are you using the SwissCovid App?*** | | | | | - Yes, permanently (Please go directly to 10.2**.**) - Yes, but sometimes I turn off Bluetooth to pause the SwissCovid app (Please go directly to 10.2**.**) - No, but I’m planning to use it (Please go directly to 10.3**.**) - No (Please go directly to 10.3**.**) | | | |
| ***10.2 Were you ever notified by the SwissCovid App that you have been in close proximity to a corona-positive person****)****?*** | | | | | - No, I have never received a notification - Yes, I called the recommended info line for SwissCovid - Yes, I undertook other steps   *Please specify:* __________________________   - Yes, but I did not undertake any steps | | | |
| ***10.3 Why are you currently not using the SwissCovid App?*** | | | | | - I have not heard about the app - I don’t think the app is useful for me - I can’t install the app (e.g. owing to technical difficulties or because I do not own an Android or iOS smartphone) - I fear for my privacy and protection of my data - Other reasons - *Please specify:* __________________________ | | | |
|  | | | | | | | | |
| ***What motivates you to be a part of Corona Immunitas? What is your experience with our study?***  ____________________________________________________________________________________  ____________________________________________________________________________________ | | | | | | | | |

**Supplementary Material Table S1.** Administrative details of each study center (Corona Immunitas, Switzerland 2020-2021)

| **Study sites, city (website)** | **Principal investigator(s)** | **Companies, institutes and laboratories involved** | **Funding sources (private/public)** |
| --- | --- | --- | --- |
| **Geneva University Hospitals, Geneva** (<https://www.hug.ch/medecine-premier-recours/unite-epidemiologie-populationnelle>) | Prof. Idris Guessous, MD PhD Dr. Silvia Stringhini, PhD  Prof. Antoine Flahault, MD PhD | - HUG, Division of Laboratory Medicine - Geneva Center for Emerging Viral Diseases and Laboratory of Virology - Institute of Global Health, University of Geneva | - Swiss FOPH (public) - SSPH+ (private) - Fondation de Bienfaisance du Groupe Pictet (private) - Fondation Ancrage (private) - Fondation Privée des HUG (private) - Center for Emerging Viral Diseases (public) |
| **Unisanté, Lausanne** (<https://www.unisante.ch/fr>) | Prof. Valérie D'Acremont Prof. Murielle Bochud  Dr. Semira Gonseth Nusslé | - Office du Médecin Cantonal Dr. Eric Masserey, Médecin cantonal adjoint, Direction Générale de la Santé Vaud - Microbiology laboratory Prof. Gilbert Greub, médecin chef, directeur & chef de service, Laboratoires de microbiologie, CHUV - Biobanking laboratory Prof. Jacques Fellay, médecin adjoint & responsable d'unité, Médecine de précision, CHUV - Public health pediatrics Dr. Olivier Duperrex, référent en pédiatrie communautaire, Unisanté, département promotion de la santé et préventions - Social sciences Dr. Nolwenn Bühler, Chercheuse FNS senior, Institut des sciences sociales, Université de Lausanne - Swiss biobanking platform, Dr. Christine Currat, Executive Director - Coordination with asylum seekers centers : Prof. Patrick Bodenmann, médecin chef & chef de département, Unisanté, département vulnérabilités & médecine sociale - Coordination with dispatched centers (Rennaz, Yverdon, Bus Santé) : Myriam Pasche, cadre de direction, Unisanté - Prestations en promotion de la santé | - Center for Primary Care and Public Health, (Unisanté), University of Lausanne (public) - The Leenaards Foundation (private) - The Foundation for the University of Lausanne (public) - Direction Générale de la Santé du Canton de Vaud (public) - SSPH+ (private) |
| **Population Health Laboratory, Fribourg** (<https://www3.unifr.ch/med/de/research/groups/pophealthlab>) | Prof. Arnaud Chiolero, MD PhD  Dr. Stéphane Cullati, PhD  Dr. Pierre-Yves Rodondi, MD | - MedHome: organisation providing study nurses for at home visits (https://www.med-home.ch/) - Blood collection sites: 3 Cantons Hospital of Fribourg (HFR) sites (HFR Meyriez-Murten, HFR Tafers, HFR Riaz), 2 private medical center sites (Permanence médicale de Fribourg, Centre médical de la Veveyse) - Central laboratory of the cantonal hospital, under Dr Jeal-Luc Magnin and Dr Stefan Pfister (https://www.h-fr.ch/nos-specialites/nos-specialites-medicales/laboratoire) - Blood sample transport: RollExpress (h[ttp://www.rollexpress.ch/](http://www.rollexpress.ch/))   Institute of Family Medicine, Department of Community Health, University of Fribourg, Prof Pierre-Yves Rodondi | - Swiss FOPH (public) - University Fribourg (public) - SSPH+ (private) |
| **Swiss TPH, Basel** ([www.swisstph.ch](http://www.swisstph.ch)) | Prof. Nicole Probst-Hensch, Dr. phil. II et PhD  Dr. Medea Imboden, PhD  Prof. Dr. Daniel Paris | - Prof. Dr. Jürg Utzinger, Director Swiss TPH - Cantonal Doctor BS - Health Department BL - PD Dr. Mio Savic, Universitätsspital Basel - Dr. Carlos Quinto FMH/Canton BL - Prof. Dr. Manuel Battegay, Universitätsspital Basel | - Swiss TPH (public) - SSPH+ (private) - Canton BS and Canton BL (public) |
| **Cantonal Medical Service, Neuchâtel** ([www.ne.ch/quidcovid](http://www.ne.ch/quidcovid)) | Dr. Laurent Kaufmann, MD |  | - SSPH+ (private) |
| **Cantonal hospital and Children’s Hospital of Eastern Switzerland, St. Gallen & Grisons** ([www.infekt.ch](http://www.infekt.ch)) | PD Dr. Philipp Kohler, MD, MSc  Dr. Christian Kahlert, MD | - Clinic for Infectious Diseases and Hospital Epidemiology,  Cantonal Hospital St. Gallen - Children’s Hospital of Eastern Switzerland - Medical Research Centre, Cantonal Hospital St. Gallen, - University Children’s Hospital Zürich, labormedizinisches zentrum Dr. Risch, Schaan | - Swiss FOPH (public) - SNSF (public) - Canton SG (public) - SSPH+ (private) |
| **Institute of Public Health and Department of Business Economics, Health & Social Care, Lugano** (<https://search.usi.ch/en/organisational-units/501/institute-of-public-health>) | Prof. Emiliano Albanese, MD, PhD Prof. Luca Crivelli, PhD | - IPH, USI - DEASS, SUPSI - Istituto di ricerca in biomedicina (IRB) - University of Applied Sciences & Arts of Southern Switzerland – SUPSI - Ente Ospedaliero Cantonale Ticino (EOC) | - Swiss FOPH (public) - SSPH+ (private) - USI (public) - SUPSI (public) |
| **Epidemiology, Biostatistics and Prevention Institute, Zurich (**[www.ebpi.uzh.ch/](http://www.ebpi.uzh.ch/)) | Prof. Dr. Milo A. Puhan, MD, PhD  Prof. Dr. Jan Fehr, MD  Dr. Anja Frei, PhD | - EBPI, UZH - Zentrum für Reisemedizin, UZH | - Swiss FOPH (public) - SSPH+ (private) - UZH (public) - UZH Foundation (private) |
| **Valaisan Health Observatory and Central Institute of Hospitals, Hospital of Valais, Sion, VS** ([www.ovs.ch](http://www.ovs.ch) <https://www.hopitalduvalais.ch/fr/accueil.html>) | Prof. Arnaud Chiolero, MD PhD  Luc Fornerod  Dr. Frank Bally, MD  Delphine Berthod, MD MPH  Alexis Dumoulin, PhD  Prof. Nicolas Troillet, MD MSc | - Observatoire valaisan de la santé, Sion, VS  - Institut central des hôpitaux, Hôpital du Valais, Sion, VS | - SSPH+ (private) - Observatoire valaisan de la santé, Sion, VS (public) - Institut central des hôpitaux, Hôpital du Valais, Sion, VS (public) |
| **Institute of Primary Health Care (BIHAM), BE (**<https://www.biham.unibe.ch/research/index_eng.html>) | Nicolas Rodondi, MD, MAS |  | - SSPH+ (private) |
| **Clinical Trial Unit (CTU), Luzern Cantonal Hospital, LU (**<https://www.luks.ch/ihr-luks/lehre-und-forschung/clinical-trial-unit>**)** | Irène Frank, Dr.Sc |  | - SSPH+ (private) |

HUG, Geneva University Hospitals; FOPH, Federal Office of Public Health; SSPH+, Swiss School of Public Health; TPH, Tropical and Public Health Institute; SNSF, Swiss National Science Foundation; IPH, Institute of Public Health; USI, University of Italian Switzerland; DEASS, Department of Business Economics, Health & Social Care; SUPSI, University of Applied Sciences & Arts of Southern Switzerland; CHUV, Vaud Central University Hospital; HFR, Fribourg Cantonal Hospital; VD, Canton Vaud; Canton Basel-City; BL, Canton Basel-Land; SG, Canton St. Gallen; BE, Canton Bern; LU, Canton Lucerne; EBPI, Epidemiology, Biostatistics and Prevention Institute; UZH, University of Zurich; CTU, Clinical Trial Unit; BIHAM, Bern Institute for Family Medicine; TBD, to be determined. (Corona Immunitas, Switzerland, 2020-2021)

**Supplementary Material Table S2.** Evaluation of Serological Tests (Corona Immunitas, Switzerland, 2020-2021)

| No | Submitted On |  | **POINTS** | **max. points** | **Comments** |
| --- | --- | --- | --- | --- | --- |
|  | **General data** |  |  |  |  |
|  | **Laboratory Company** |  |  |  |  |
|  | Contact person |  |  |  |  |
|  | Email address |  |  |  |  |
|  | Phone |  |  |  |  |
|  | **Name of the test** |  |  |  |  |
|  | Equipment/machines needed at sites |  |  |  |  |
|  | Approval by regulatory agency |  |  | 2 |  |
|  | If yes, name of the regulatory agency |  |  |  |  |
|  | CE IVD |  |  | 1 |  |
|  | Website with test specifications |  |  |  |  |
| **1** | **Test principle** |  |  |  |  |
|  | Describe which technical/methodological approach was chosen |  |  | 1 |  |
| **2** | **Type of target antigens that are used** |  |  |  |  |
|  | S1/S2/N |  |  | 3 |  |
| **3** | **Total antibody classes that can be detected** |  |  |  |  |
|  | List antibody classes |  |  | 4 |  |
| **4** | **Quantitative read-out** |  |  |  |  |
|  | IgG quantitative |  |  | 2 |  |
| **5** | **Data about "neutralization"** |  |  |  |  |
|  | Can the test give any information about neutralizing antibodies? |  |  | 2 |  |
| **6** | **Minimally required volume** |  |  |  |  |
|  | How many μl Serum needed to run the test? |  |  | 2 |  |
| **7** | **Requirements for second tests** |  |  |  |  |
|  | Is any confirmatory assay needed? |  |  | 2 |  |
|  | Describe the need for a second test (e.g. in case of low positivity and / or values just below the threshold of positivity, all positive, inconclusive results, additional S1 testing) |  |  |  |  |
|  | Is there already a second test in place from the same company / laboratory? |  |  |  |  |
| **8** | **Test accuracy for population based studies** |  |  |  |  |
|  | Specificity: Estimates (e.g. blood donors, healthy volunteers vs. past human coronavirus infections and autoimmune diseases) and provide information on cross reactivity |  |  | 4 |  |
|  | Sensitivity: Estimates (correlate to RT-PCR documented cases, with delay between symptoms & collection of the serum, i.e 5-9 days, 10-14 days. If possible, indicate, case mix of tested inpatient and outpatient) |  |  |  |  |
|  | Estimation of the positive predictive value in a low and very low prevalence population |  |  |  |  |
|  | Proportion of inconclusive results |  |  |  |  |
| **9** | **Validation studies** |  |  |  |  |
|  | Number of validated studies |  |  | 3 |  |
|  | Of which are independently validated |  |  |  |  |
|  | Validated in Switzerland? |  |  |  |  |
|  | If yes, by which laboratory |  |  |  |  |
|  | Populations included in validation studies |  |  |  |  |
|  | References: DOI Numbers |  |  |  |  |
| **10** | **Standardization of testing across sites** |  |  |  |  |
|  | Quantification |  |  | 3 |  |
|  | Ring trials |  |  |  |  |
|  | Describe infrastructure and training needs for laboratories at study sites |  |  |  |  |
| **11** | **Availability at all sites** |  |  |  |  |
|  | Describe how test kits and equipment are made available to all study sites by Mid / End May 2020 |  |  | 4 |  |
|  | Describe if and how the supply for around 60’000 kits can be met between now and April 2021. Further, describe the location of production sites, for all parts as machine and kits |  |  |  |  |
| **12** | **Cost per test** |  |  |  |  |
|  | Costs per test |  |  | 4 |  |
|  | Overall cost per test (including laboratory cost) |  |  |  |  |
| **13** | **Logistics** |  |  |  |  |
|  | What do you provide to simplify the work at each center? |  |  | 2 |  |
| **14** | **Maintenance** |  |  |  |  |
|  | How is maintenance secured and what is the respond time in case of any test-related problems? |  |  | 2 |  |
| **15** | **Experience to date** |  |  |  |  |
|  | Where is your test already running (sites, countries)? |  |  | 3 |  |
|  | Can you indicate 2-3 referee? |  |  |  |  |
| **16** | **Possible in kind** |  |  |  |  |
|  | Possible in kind eg test kits and other contributions for Corona Immunitas CI |  |  | 6 |  |
| **17** | **Strength limitation of your test** |  |  |  |  |
|  | Why should we choose your test? |  |  | 2 |  |
|  | What is your unique selling proposition (USP)? |  |  |  |  |
|  | What are the limitations of your test? |  |  |  |  |
|  | Gaps? |  |  |  |  |
| **18** | **Motivation to submit this offer** |  |  |  |  |
|  | Short explanation why you offer |  |  | 2 |  |
| **19** | **Other** |  |  |  |  |
|  | Anything else you want to add? |  |  |  |  |
|  | **List of annexes** |  |  |  |  |
|  | Reports, publications, etc. |  |  |  |  |
|  |  |  |  |  |  |
|  |  | **Grand total** | **0** | **points of max. 54** | |

(Corona Immunitas, Switzerland, 2020-2021)
